# Nanomaterial-based CT contrast agents and their applications in image-guided therapy

**DOI:** 10.7150/thno.79625

**Published:** 2023-01-01

**Authors:** Zeyu Jiang, Meihua Zhang, Peifeng Li, Yin Wang, Qinrui Fu

**Affiliations:** 1 Institute for Translational Medicine, The Affiliated Hospital of Qingdao University, College of Medicine, Qingdao University, Qingdao 266021, China.; 2 Key Laboratory of Birth Regulation and Control Technology of National Health Commission of China, Maternal and Child Health Care Hospital of Shandong Province affiliated to Qingdao University, Jinan, 250014, China.; 3 Department of Cardiovascular Medicine, The Affiliated Hospital of Qingdao University, College of Medicine, Qingdao University, Qingdao 266021, China.

**Keywords:** material science, contrast agent, CT imaging, diagnosis, therapy

## Abstract

Computed tomography (CT), a diagnostic tool with clinical application, comprehensive coverage, and low cost, is used in hospitals worldwide. However, CT imaging fails to distinguish soft tissues from normal organs and tumors because their mass attenuation coefficients are similar. Various CT contrast agents have been developed in recent years to improve the sensitivity and contrast of imaging. Here, we review the progress of nanomaterial-based CT contrast agents and their applications in image-guided therapy. The CT contrast agents are classified according to their components; gold (Au)-based, bismuth (Bi)-based, lanthanide (Ln)-based, and transition metal (TM)-based nanomaterials are discussed. CT image-guided therapy of diseases, including photothermal therapy (PPT), photodynamic therapy (PDT), chemotherapy, radiotherapy (RT), gas therapy, sonodynamic therapy (SDT), immunotherapy, starvation therapy, gene therapy (GT), and microwave thermal therapy (MWTT), are reviewed. Finally, the perspectives on the CT contrast agents and their biomedical applications are discussed.

## Introduction

X-ray computed tomography (CT) is one of the most widely and commonly used non-invasive clinical imaging modalities owing to its high efficiency, low cost, and easy access 1. The CT scanner was developed by Godfrey Hounsfield at Electric and Musical Industries (EMI) LTD, and the first clinical trial was performed using a prototype CT scanner (Mark I) in 1972 2. CT imaging can precisely display human tissues and organs, significantly advancing diagnostics. Therefore, Godfrey Hounsfield was awarded the Nobel Prize in Medicine in 1979, and the CT unit was named Hounsfield unit (HU) after him to honor his contribution to science 3.

When an X-ray beam traverses a patient's body, it is attenuated by absorption or deflection 4. The detectors, located on opposite sides of the patient, collect the attenuated X-rays, which are then converted and computerized to obtain images 5. This X-ray attenuation (mass attenuation coefficient) is related to the atomic number (Z) and K-shell absorption edge (K-edge) energy (*i.e.*, the energy required to eject an inner K-shell electron) of the elements in tissues or contrast agents 6. When an X-ray beam passes through a human body, tissues with different attenuation coefficients can be distinguished and presented on CT images. The inherent contrast between the bone and surrounding tissues is large enough to distinguish them. ​However, since most soft tissues, such as normal organs and tumors, have similar mass decay coefficients, it is difficult to distinguish subtle changes in soft tissues. To address this challenge, exogenous CT contrast agents with additional X-ray attenuation have been introduced at lesion sites to distinguish soft tissues, such as normal organs and tumors 7. ​Currently, commercially available CT contrast agents for *in vivo* imaging are composed of iodinated small molecules, including meglumine diatrizoate, iopromide, iohexol, and iodixanol (**Table 1**) 8. However, the iodinated contrast agents suffer from several limitations: (1) small atomic number and low K-edge energy resulting in a low contrast efficiency, (2) limited applicable population due to its allergic reaction and nephrotoxicity, and (3) non-specific biodistribution requiring high concentrations to improve contrast. Therefore, developing CT contrast agents with superior efficiency, safety, and excellent targeting has become an urgent and challenging task to improve the contrast effect of CT imaging.

Nanotechnology, a transformative technology with the potential to stimulate scientific innovation while greatly benefiting society, has come a long way in the last few decades. Various nanomaterials have been developed as promising agents for biomedical applications due to their unique physical and chemical properties, such as controllable synthesis and easy surface modification 9, 10. Among the diverse development of nanomaterials, metal-based inorganic nanoparticles (NPs) with high atomic numbers and large X-ray attenuation coefficients show great potential for achieving specific bioimaging as CT contrast agents 11. To date, many contrast agents for CT imaging have been reported, including gold (Au)-based nanomaterials, bismuth (Bi)-based nanomaterials, lanthanide (Ln)-based nanomaterials, transition metal (TM)-based nanomaterials, among others.

The separation of diagnosis and treatment is one of the main challenges in the clinic. Advanced nanomaterials with integrated diagnostic and therapeutic capabilities can provide direct evidence of an early diagnosis, development, and progression of the disease, enabling real-time imaging of drugs for disease detection and image-guided treatment. Therefore, multifunctional nanomaterials with integrated diagnostic and therapeutic properties have emerged as a research hot spot.

**Figure 1:**
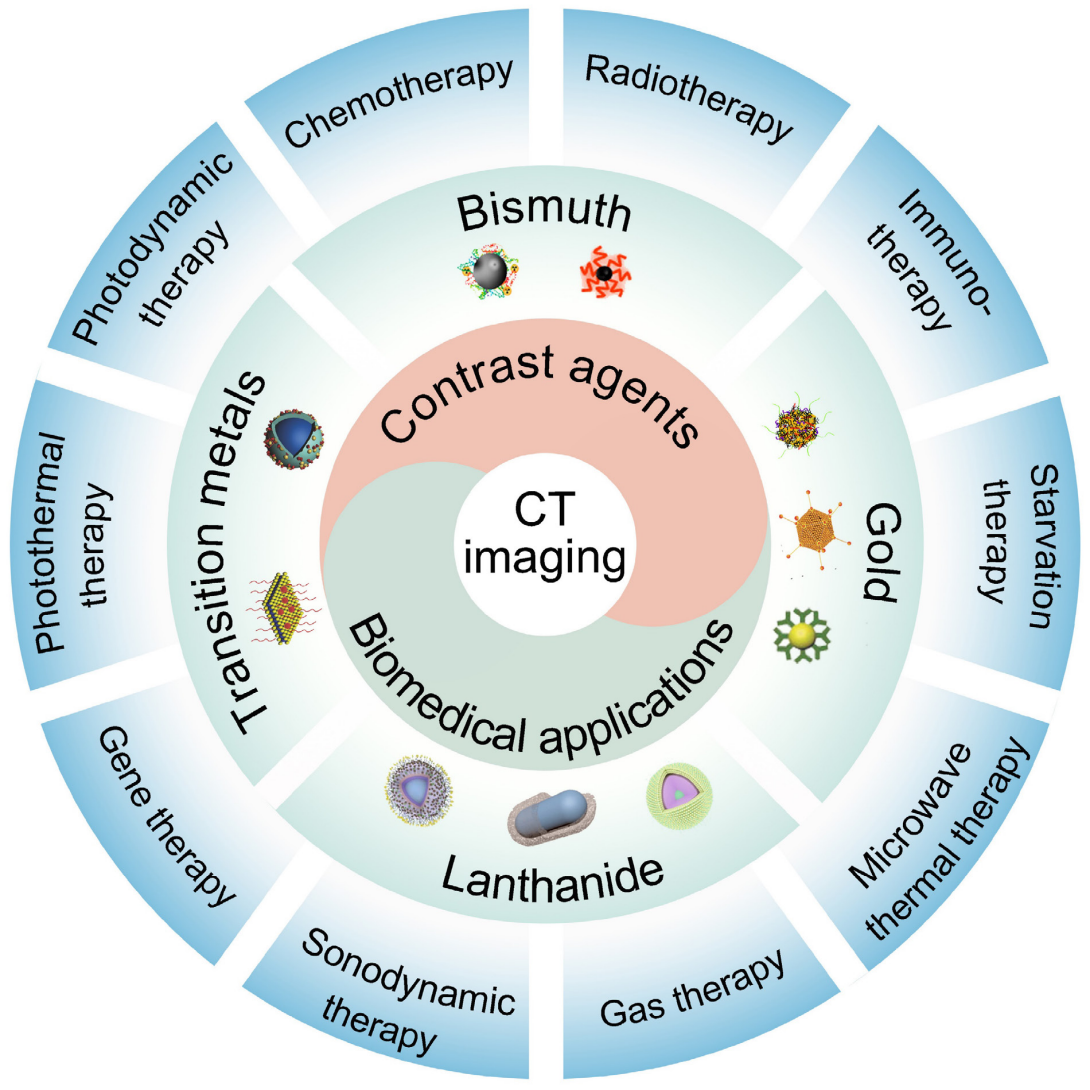
Schematic illustration of nanomaterial-based CT contrast agents and CT image-guided therapies.

Here, we focus on the development of nanomaterial-based CT contrast agents and their biomedical applications in recent years (**Figure 1**). First, we offer a comprehensive outline of the available CT contrast agents, including Au-based, Bi-based, Ln-based, and TM-based nanomaterials. Particular attention has been paid to the synthesis methods and imaging capabilities of these contrast agents. We then address recent developments in CT image-guided therapy, including photothermal therapy (PPT), photodynamic therapy (PDT), chemotherapy, radiotherapy (RT), gas therapy, sonodynamic therapy (SDT), immunotherapy, starvation therapy, gene therapy (GT), and microwave thermal therapy (MWTT). Challenges, opportunities, and future research priorities are then discussed.

## Nanomaterial-based CT contrast agents

### Gold nanoparticles

Iodinated CT contrast agents, widely used in clinical diagnosis, are rapidly excreted through the kidney, resulting in a short imaging time and nephrotoxicity. In addition, iodinated contrast agents are distributed nonspecifically throughout the intravascular and extravascular space, resulting in ambiguous CT images 12. Gold nanoparticles (AuNPs), with high X-ray attenuation and K-edge energy (80.7 keV), can provide higher imaging contrast at high X-ray tube voltages than iodinated CT contrast agents at the same concentration 8. AuNPs also allow facile surface modifications to increase their biocompatibility and durability due to their high affinity for thiol derivatives 13. Furthermore, AuNPs with controllable shapes have been synthesized as nanospheres 14, 15, nanorods 16-19, nanostars 20-22, nanoplates 23, nanocages 24, nanoshells 25, nanoprisms 26, and nanodisks 27. These AuNPs with unique chemical, electrical, and optical properties have been widely used in the biomedical field.

**Figure 2:**
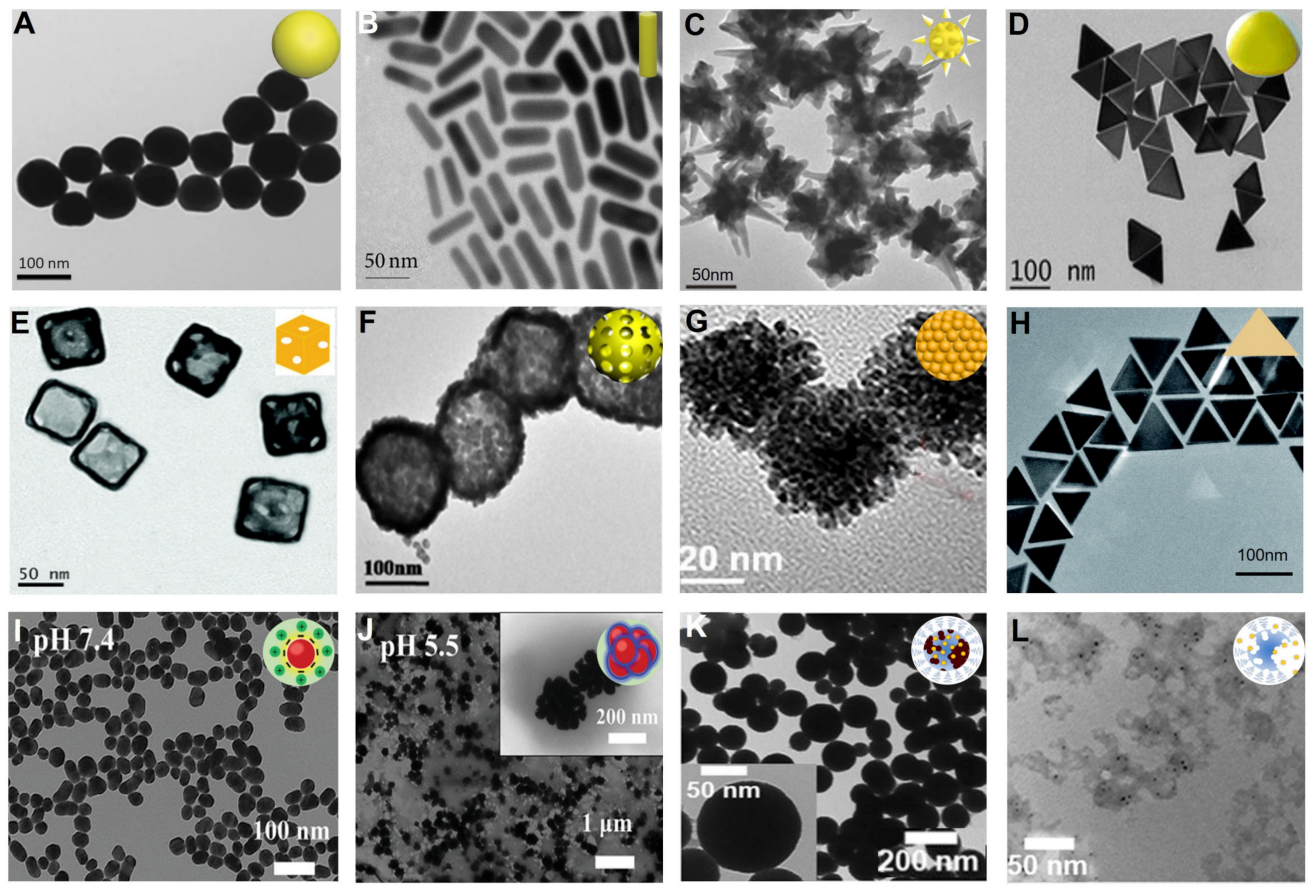
Gold-based NPs. TEM images of **(A)** gold nanospheres, **(B)** gold nanorods, **(C)** gold nanostars, **(D)** gold nanoplates, **(E)** gold nanocages, **(F)** gold nanoshells, **(G)** Au_2_Pt NPs, **(H)** Gd-Au NPrs, **(I)** CPP-PSD@Au at pH 7.4, **(J)** CPP-PSD@Au at pH 5.5, **(K)** PPB NPs, **(L)** PPB NPs in 10 mM H_2_O_2_. **(A)** Adapted with permission from 29, Copyright 2019 Springer Nature. **(B)** Adapted with permission from 32, Copyright 2016 Hindawi Publishing Corporation. **(C)** Adapted with permission from 22, Copyright 2016 Wiley-VCH. **(D)** Adapted with permission from 23, Copyright 2018 American Chemical Society. **(E)** Adapted with permission from 24, Copyright 2018 The Royal Society of Chemistry. **(F)** Adapted with permission from 25, Copyright 2017 American Chemical Society. **(G)** Adapted with permission from 43, Copyright 2020 Elsevier. **(H)** Adapted with permission from 26, Copyright 2017 The Royal Society of Chemistry. **(I-J)** Adapted with permission from 48, Copyright 2021 Wiley-VCH. **(K-L)** Adapted with permission from 49, Copyright 2019 American Chemical Society.

It has been shown that the AuNP size does not affect the X-ray attenuation at the same concentration 28, 29. But an enhanced *in vivo* CT imaging signal can be achieved by increasing local intratumoral concentrations that can be achieved by designing appropriately sized particles to aggregate into the region of interest 29, 30. For example, Dong *et al*. synthesized six PEGylated gold nanospheres (AuNSps) with different sizes in the range of 4-152 nm and evaluated their CT contrast properties *via* clinical CT scanners (**Figure 2A**) 29. The results showed that small AuNSps (4 nm and 15 nm) produced more long-lasting and stronger CT contrast than large ones in the blood vessels as they were not easy to be recognized and cleared by the mononuclear phagocyte system when passing through the liver and kidney, resulting in a high concentration of AuNSps enriched in the blood pool 31. Conversely, large AuNSps (>50 nm) were easily deposited in the liver and spleen, offering outstanding CT contrast of the liver and spleen region.

Although the CT contrast effect *in vitro* is not substantially affected by the shape of NPs, the gold nanorods (AuNRs) exhibit a stronger *in vivo* CT contrast due to better performance in evading clearance by phagocytes and attaining a longer circulation time and higher intratumoral enrichment than AuNSp 16-19. Liang *et al.* developed Arg-Gly-Asp (RGD)-modified AuNR nanoprobes with no toxicity, high contrast, and long imaging time as promising contrast agents for *in vivo* CT imaging (**Figure 2B**) 32. Also, gold nanostars (AuNSs) with multiple sharp branches can be modified with polydopamine-targeting peptide to improve CT contrast due to the greater surface area and extended serum half-life, achieving much longer intravascular signal stability (**Figure 2C**) 20-22. Moreover, Lu *et al.* developed a P75 (a novel EGFR-targeting peptide)-modified triangular gold nanoplates (AuNPTs-PEG-P75) that could target overexpressed epidermal growth factor receptor (EGFR) in non-small cell lung cancer to realize specific CT imaging *in vivo* (**Figure 2D**) 23.

Theranostic agents represent an emerging method to meet clinical needs by combining diagnostic and therapeutic modalities into one specific system. As inorganic nanoscale carriers, gold nanocages (AuNCs) with hollow structures and gold nanoshells (AuNShs) with porous walls have been investigated as theranostic agents over the past few years 33-35. The surfaces of AuNCs and AuNShs can be easily modified by Au-S bonding with various molecules and ligands, making them promising drug delivery vehicles and CT contrast agents 36, 37. Hu *et al.* developed AuNC theranostic agents by integrating a CT contrast agent, doxorubicin (DOX), and miR-122 (a liver-specific miRNA) into a single system for achieving CT image-guided enhanced cancer therapy by combining chemotherapy and gene therapy (**Figure 2E**) 24. Li *et al.* designed bovine serum albumin (BSA)-coated hollow AuNShs as a multifunctional drug delivery platform to achieve CT image-guided drug delivery (**Figure 2F**) 25.

Despite the advantages of CT imaging in spatial resolution, accurate differentiation of different tissue densities remains a challenge. The use of multimodal imaging contrast agents is a promising avenue for future innovations in biomedical research, which can help overcome the inadequacies of a single CT imaging modality, achieve complementary advantages, and broaden the range of applications of molecular imaging techniques 38-41. As one of the noble metal NPs, AuNPs possess excellent light absorption and photoacoustic (PA) imaging capability due to the localized surface plasmon resonance (LSPR) effect 42. Thus, Xing *et al.* fabricated a bimetallic nanoplatform (Au_2_Pt NPs) with strong absorption in the NIR region and high X-ray attenuation with excellent CT/PA dual-modal imaging capability (**Figure 2G**) 43. The CT value at the tumor site increased from 19.06 Hu to 489.35 HU 12 h after injection, and a strong PA signal could be observed, improving the diagnostic ability by combining the PA and CT imaging. The introduction of magnetic resonance imaging (MRI) can effectively compensate for the limitation of CT imaging in distinguishing soft tissues 44-46. Lu *et al.* prepared multifunctional gadopentetic acid-decorated gold nanoprisms (Gd-AuNPrs), in which gadolinium with excellent magnetic properties and AuNPs with high X-ray attenuation were used for *T*_1_-weighted MRI and CT imaging, respectively (**Figure 2H**) 26. The longitudinal relaxivity (r_1_) of Gd-AuNPrs was 23.1 mM^-1^ s^-1^, and its X-ray absorption coefficient was 959.3 HU L g^-1^; hence the Gd-AuNPrs could be used as promising dual-modal imaging contrast agents.

Tumor microenvironment (TME) such as pH, hypoxia, and H_2_O_2_-responsive assembly of NPs can improve the accumulation of contrast agents in tumors, achieving enhanced CT contrast 47. Zhang *et al.* developed a pH-sensitive Au-based nanotracer (CPP-PSD@Au) that was sequentially modified with the pH-sensitive polymer polysulfonamide (PSD) and cell-penetrating peptide (CPP) for enhanced labeling and long-term tracking of CT imaging of stem cells in pulmonary fibrosis treatment (**Figure 2I-J**) 48. Once the nanotracer entered the cell, the CPP was shed from the surface of the CPP-PSD@Au in the endosome, causing the monodisperse CPP-PSD@Au to rapidly aggregate into agglomerates due to the PSD protonation in the acidic environment, thereby significantly increasing intracellular retention for enhanced CT imaging contrast. However, large nanocomposites are not readily metabolized in the cells, which may lead to increased cytotoxicity. Thus, Cormode *et al.* developed H_2_O_2_-responsive CT and PA dual-mode contrast agents using the AuNP payload in an arylboronate polyphosphazene derivative (PPB) core (**Figure 2K**) 49. PPB was efficiently degraded in the TME with overexpression of H_2_O_2_, which triggered a switch-off of the PA signal and a slight reduction in the CT signal, causing a concentration reduction of local AuNPs (**Figure 2L**) 50. This H_2_O_2_-triggered degradation of the nanoprobes led to a significant increase in the CT/PA contrast ratio, allowing ratiometric imaging to identify reactive oxygen species (ROS) overexpression.

### Bismuth-based nanoparticles

**Figure 3:**
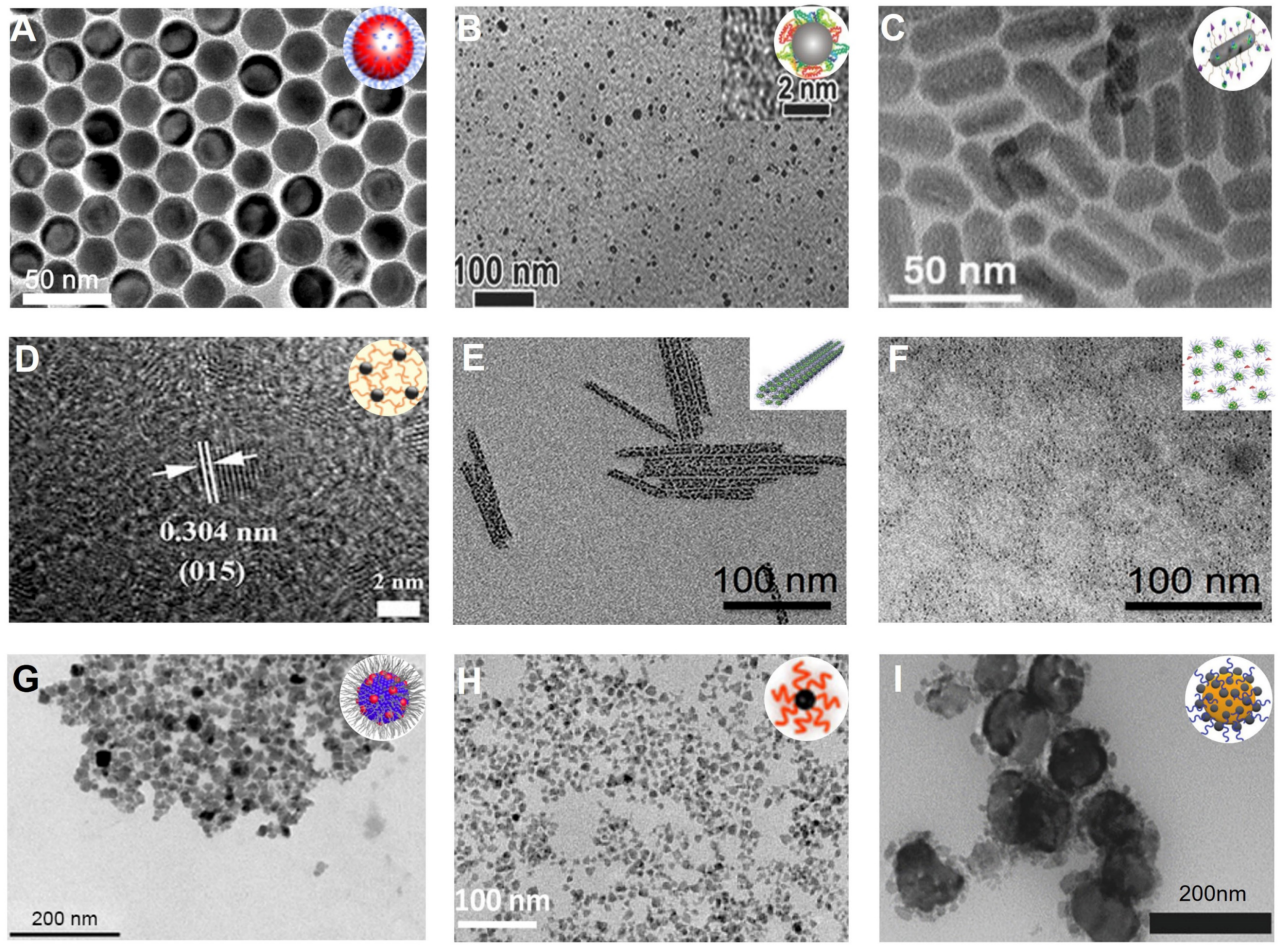
Bismuth-based NPs. TEM images of **(A)** BiNPs, **(B)** Bi_2_S_3_ NPs, **(C)** Bi_2_S_3_ nanorods, **(D)** Bi_2_Se_3_ nanodots,** (E)** (BiO)_2_CO_3_ nanotubes, **(F)** (BiO)_2_CO_3_ nanoclusters, **(G)** HA-Bi_2_O_3_ NPs, **(H)** Cu_3_BiS_3_ NDs, **(I)** BiOI@Bi_2_S_3_ NPs. **(A)** Adapted with permission from 54, Copyright 2016 American Chemical Society. **(B)** Adapted with permission from 58, Copyright 2016 Wiley-VCH. **(C)** Adapted with permission from 59, Copyright 2019 Wiley-VCH. **(D)** Adapted with permission from 62, Copyright 2021 Frontiers Media S.A. **(E-F)** Adapted with permission from 63, Copyright 2018 American Chemical Society. **(G)** Adapted with permission from 64, Copyright 2017 Dove Press Ltd. **(H)** Adapted with permission from 65, Copyright 2016 American Chemical Society. **(I)** Adapted with permission from 66, Copyright 2017 Wiley-VCH.

Bismuth (Bi) possesses a higher atomic number (Bi: 83, Au: 79) and larger X-ray attenuation coefficient (Bi: 5.74 cm^2^/g, Au: 5.16 cm^2^/g, at 100 keV) than Au, and is a promising CT contrast agent for diagnosis of diseases 51. As a noble metal, the high cost of Au inevitably limits its applications in clinical practice. However, Bi is a relatively cheap and low-toxic heavy metal and has been used as a pharmaceutical ingredient to treat various diseases, such as gastritis, dyspepsia, ulcers, and infections 52. Bi nanoparticles (BiNPs) can improve the X-ray absorption efficiency and address the bottleneck of CT imaging contrast agents in terms of sensitivity 53. Wei *et al.* synthesized dodecanethiol-modified BiNP contrast agents for CT imaging of the gastrointestinal tract (GIT) (**Figure 3A**); the CT contrast enhancement of the BiNP contrast agents was 1.5-fold higher than BaSO_4,_ a traditional clinical CT contrast agent for the diagnosis of GIT 54.

Bi-based chalcogenides are also commonly used as CT contrast agents in clinical use due to their stable chemical properties 55. In 2006, Rabin and coworkers developed polyvinylpyrrolidone (PVP)-coated bismuth sulfide (Bi_2_S_3_) nanocrystals as an injectable CT contrast agent 56, which possessed higher stability and X-ray attenuation coefficient (5-fold higher than an iodinated contrast agent), longer circulation times *in vivo* (140 ± 15 min), and more reliable security than iodinated CT contrast agents. The lymph nodes could be distinctly observed, and the CT signal intensity in the liver region increased from -22 ± 77 HU to 740 ± 210 HU at 24 h post-administration, indicating that Bi_2_S_3_ NPs could be used as an excellent CT contrast agent for diagnosis of diseases.

​Besides the X-ray attenuation coefficient, the circulation half-life is one of the essential parameters that must be considered to improve the CT imaging capability of Bi-based contrast agents. In this context, modification with biocompatible materials may improve the circulating half-life of Bi-based contrast agents. For example, the circulation half-life of Pluronic F127-modified Bi_2_S_3_ NPs could be extended to 5 h, demonstrating excellent CT contrast capability *in vivo* 57. Chai *et al.* prepared BSA-modified Bi_2_S_3_ contrast agent with high circulation half-life (14.85 h), X-ray attenuation, and 6-fold higher CT imaging capability than iobitridol (**Figure 3B**) 58. Prolonging the half-life of contrast agents in tumor cells has prominent clinical significance. Zhang *et al.* prepared CPP-modified Bi_2_S_3_ nanorods (NRs) CT contrast agent (BPZP) by a solvothermal method to extend the contrast effect at the tumor site (**Figure 3C**) 59. The 4T1 tumor-bearing mice were given BPZP intravenously, and the CT signals at the tumor site were observed at 2 h post-injection and maintained at 12 h post-injection, which was attributed to the persistent escape of BPZP under CPP action.

Targeted imaging is also an effective method to enhance the imaging capabilities of specific organs, especially tumors. Zhou *et al.* prepared folate-modified perfluorohexane (PFH) NPs to carry Bi_2_S_3_ nanodots for targeted CT imaging of the tumor 60. After intravenous administration, the CT values of tumors in the targeted group (251 HU) was enhanced compared with the non-targeted group (33 HU) due to the excellent ability of folate to target tumors. Moreover, Kinsella *et al.* developed a nine-amino acid cyclic peptide (LyP-1, CGNKRTRGC)-labeled Bi_2_S_3_ NPs for CT imaging of 4T1 tumors 61. The LyP-1 labeled Bi_2_S_3_ NPs preferentially accumulated in the tumor region (8.4 ± 2.1% injected dose per gram of tissue) relative to unlabeled NPs (3.2 ± 1.7% injected dose per gram of tissue) due to excellent targeting ability of LyP-1 for 4T1 cells, thus enabling acquisition of high-fidelity CT images of the tumor.

Apart from Bi_2_S_3_ NPs, other Bi-based nanomaterials, such as bismuth selenide (Bi_2_Se_3_), bismuth oxide (Bi_2_O_3_), bismuth oxyiodide (BiOI), bismuth oxychloride (BiOCl), and Cu_3_BiS_3_, could also be used as excellent CT contrast agents. Yuan *et al.* synthesized ultrasmall polyethylenimine (PEI)-decorated Bi_2_Se_3_ (PEI-Bi_2_Se_3_) nanodots* via* a facile room temperature method for CT imaging of cancer (**Figure 3D**) 62. The CT contrast of PEI-Bi_2_Se_3_ nanodots in the tumor region was twice as high as that of iobitridol at equivalent concentrations.

Ultrasmall-sized nanomaterials avoid potential toxicity due to their rapid renal clearance but are not effective for tumor targeting and CT imaging. To solve this contradiction, Ling *et al.* prepared bismuth subcarbonate ((BiO)_2_CO_3_) nanotubes (BNTs) with approximately 100 nm in length and 8 nm in diameter by assembling 1.5 nm ultrasmall (BiO)_2_CO_3_ nanoclusters (BNCs) (**Figure 3E**) 63. The blood-clearance half-life of the BNTs were 26.99 ± 0.41 h, which was about 7-fold longer than that of the BNCs (3.77 ± 1.53 h), suggesting that the BNT contrast agents offered the tumor accumulation ability to enhance CT imaging capability. Furthermore, after localization in the tumor tissue, BNTs could be disassembled into nanoclusters in an acidic TME (**Figure 3F**), providing BNTs a superior degradation capability.

To avoid the biotoxicity caused by the intrinsic potential accumulation of sulfur element, Wu *et al.* prepared hyaluronic acid (HA)-functionalized bismuth oxide NPs (HA-Bi_2_O_3_ NPs) *via* one-pot hydrothermal method for targeted CT imaging of the tumor (**Figure 3G**) 64. In VSMC, SMMC-7721, and MCF7 cells, the HA-Bi_2_O_3_ NPs exhibited negligible cytotoxicity at different concentrations. HA-Bi_2_O_3_ NPs could be specifically absorbed in cancer cells overexpressing CD44 receptors, and strong targeted CT imaging capabilities were observed. The CT value at the tumor site increased from 60 HU to approximately 200 HU 10 min after the injection.

Limited by the poor sensitivity of CT imaging in soft tissues, a high concentration of the contrast agent is usually required to achieve the imaging effect in the clinic 39. Due to the complementary advantages, dual-modal imaging is a good strategy for solving the issues. For example, CT imaging gives structural details of deep tissues, while PA imaging provides high sensitivity to soft tissues 40. Bimetallic sulfide-based hybrid nanoparticles have been developed for CT/PA imaging due to their unique optical properties. Chen* et al.* prepared the single-phased ternary bimetal sulfide nanomaterial, Cu_3_BiS_3_ NDs, with inherent high X-ray attenuation and strong NIR absorption coefficient (>10^5^ cm^-1^) for CT/PA dual-modal imaging (**Figure 3H**) 65. When Cu_3_BiS_3_ NDs were intravenously injected into 4T1 tumor-bearing mice at a low concentration (200 μL, 10 mg/mL), CT/PA signals of tumor were observed at 2 h post-injection and lasted for up to 24 h, indicating the excellent imaging effect of Cu_3_BiS_3_ NDs in the tumor region. Zhao *et al.* synthesized semiconductor heterojunction BiOI@Bi_2_S_3_ NPs (SHNPs) *via* the hydrothermal procedure and anion exchange method as a dual-modal contrast agent for CT/PA imaging (**Figure 3I**) 66. The BiOI@Bi_2_S_3_ heterojunction improved the absorption in the NIR window and enhanced the X-ray attenuation due to the iodine element doping. At the same concentration, SHNPs showed a higher CT signal than iopromide, while PA signals of SHNPs were also observed at very low concentrations, demonstrating the promising potential of SHNPs in CT/PA imaging.

### Lanthanide-based nanoparticles

Lanthanide (Ln) elements exhibit higher X-ray attenuation coefficients than iodine elements 67, and their K-edge energy is close to the average energies (80-120 keV) of other contrast agents used in CT imaging and can be used as CT contrast agents 12. Ln elements with atomic numbers ranging from 57 to 71 include Lanthanum (La), Cerium (Ce), Praseodymium (Pr), Neodymium (Nd), Promethium (Pm), Samarium (Sm), Europium (Eu), Gadolinium (Gd), Terbium (Tb), Dysprosium (Dy), Holmium (Ho), Erbium (Er), Thulium (Tm), Ytterbium (Yb), and Lutecium (Lu).

**Figure 4:**
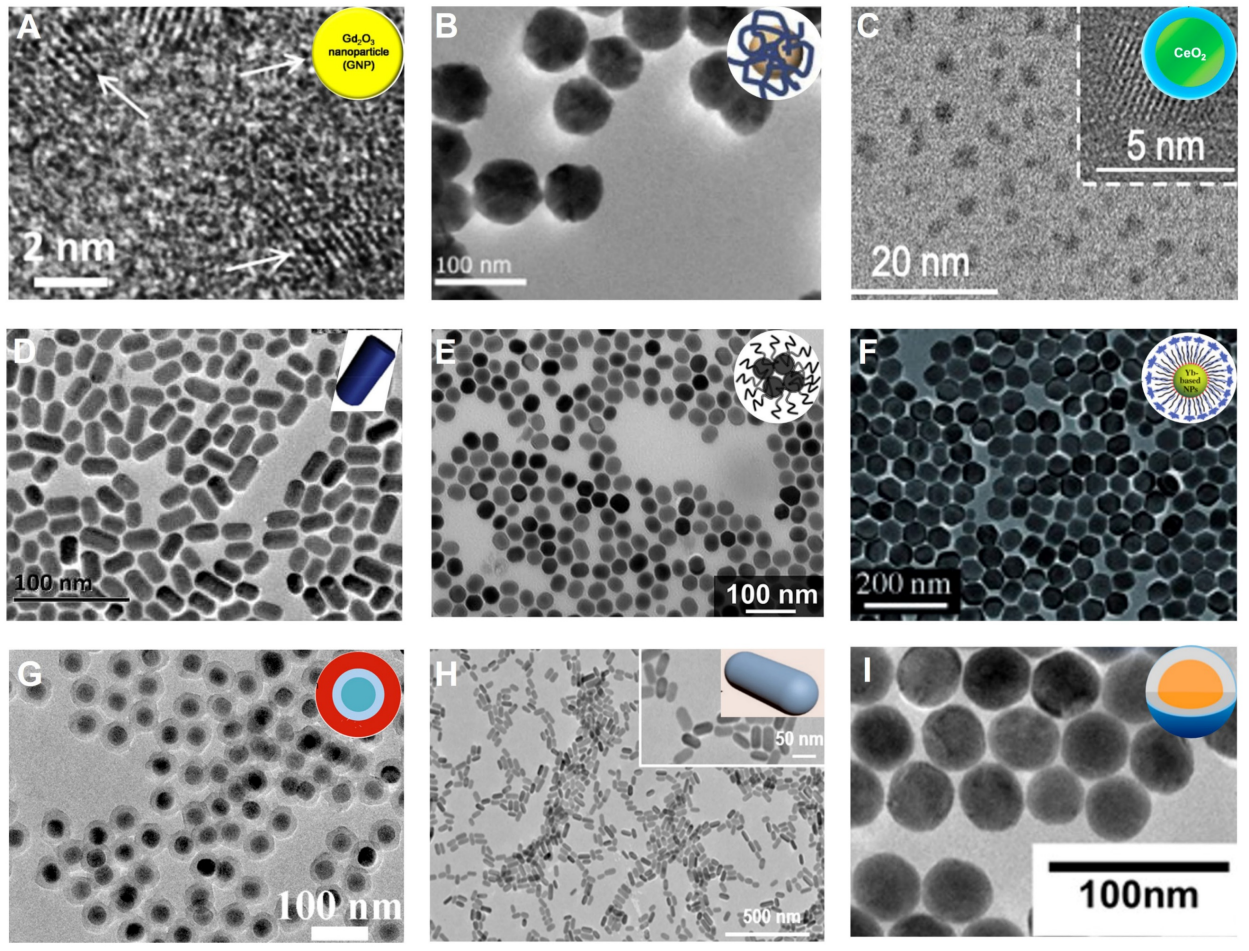
Lanthanide-based NPs. TEM of **(A)** Gd_2_O_3_ NPs, **(B)** EuO_2_ NPs, **(C)** CeO_2_ NPs, **(D)** NaHoF_4_ NPs, **(E)** NaErF_4_ NPs, **(F)** NaYbF_4_ NPs, **(G)** NaYF_4_:Nd^3+^@NaLuF_4_ NPs, **(H)** NaCeF_4_:Gd,Tb ScNPs, **(I)** Yb/Er@Lu@Gd NPs. **(A)** Adapted with permission from 68, Copyright 2019 Elsevier. **(B)** Adapted with permission from 70, Copyright 2021 IOP Publishing. **(C)** Adapted with permission from 72, Copyright 2020 American Chemical Society. **(D)** Adapted with permission from 74, Copyright 2015 Elsevier. **(E)** Adapted with permission from 76, Copyright 2018 American Chemical Society. **(F)** Adapted with permission from 78, Copyright 2012 Wiley-VCH. **(G)** Adapted with permission from 83, Copyright 2017 American Chemical Society. **(H)** Adapted with permission from 85, Copyright 2020 American Chemical Society. **(I)** Adapted with permission from 86, Copyright 2017 American Chemical Society.

​The chemical properties of metallic oxide NPs are more stable under biological and atmospheric conditions than metallic NPs and are, therefore, more suitable for biomedical applications. Lanthanide oxide (Ln_2_O_3_) NPs are excellent CT contrast agents because of their high X-ray attenuation energy 68, 69. Lee *et al*. synthesized polyacrylic acid (PAA)-coated ultrasmall Gd_2_O_3_ NPs (Gd, Z = 64) by the one-pot polyol method and investigated their X-ray attenuation properties (**Figure 4A**) 68. The X-ray attenuation rate of Gd_2_O_3_ NPs was roughly estimated to be 5.9 HU/mM, which was higher than that of Ultravist (4.40 HU/mM). PAA-coated ultrasmall Gd_2_O_3_ NPs intravenously injected into mice showed that a low injection dose of Gd (~0.015 mmol) could produce a better contrast effect than Ultravist (> 0.1 mmol). Atabaev *et al*. prepared PEI-coated Eu_2_O_3_ (Eu, Z = 63) CT contrast agents using urea homogeneous precipitation protocols (**Figure 4B**) 70. The CT value of 8 HU/mM per 1 mM metal concentration for PEI@Eu_2_O_3_ was significantly higher than PAA-Gd_2_O_3_ NPs due to the higher Ln content of the urea homogeneous precipitation protocol compared to the one-pot polyol method.

Besides Gd_2_O_3_ and Eu_2_O_3_, Cerium oxide (CeO_2_) NPs could also serve as an alternative for CT contrast agents. Cerium (Ce, Z = 58) has a K-edge energy at 40.4 keV that is slightly higher than the iodine element (33.2 keV), matches well with the X-ray beams used in CT imaging, and can produce strong CT contrast 71. Using a precipitation method, Cormode *et al.* prepared a Ce-based CT contrast agent (Dex-CeNP) consisting of dextran-coated CeO_2_ NPs for GIT imaging (**Figure 4C**) 72. Dex-CeNPs provided comparable or slightly better CT contrast than iopamidol 60 min after oral administration to healthy mice, demonstrating its potential for GIT imaging. However, when DSS-induced colitis mice received Dex-CeNPs, the CT values of the large intestine significantly increased (about 50 times) compared to iopamidol due to the affinity of dextran for inflammatory sites. Also, Dex-CeNPs neutralized free radicals from ionizing radiation while protecting against oxidative damage, which was beneficial in preventing oxidative damage caused by CT examination.

Lanthanide Fluoride (NaLnF_4_), with significant chemical stability, is an excellent CT contrast agent because it can bundle numerous ions together in the form of stabilized nanoparticles 73. As heavy rare earth elements, Ho (Z = 67), Er (Z = 68), and Yb (Z = 70) have high X-ray attenuation coefficients and thus can be used as CT contrast agents. For instance, Ho possesses a higher attenuation coefficient (3.49 cm^2^/g, at 100 keV) and K-edge value (55.6 keV) than iodine, which endows Ho-based NPs with an outstanding CT imaging contrast performance. Shi *et al.* synthesized PEGylated NaHoF_4_ NPs through the thermal decomposition method for CT imaging (**Figure 4D**) 74. *In vitro* slope of the CT values of PEG-NaHoF_4_ NPs was about 42.1 HU L/g, which was considerably higher than iobitridol (16.5 HU L/g) at the standard concentration. Moreover, when NaHoF_4_ NPs were injected intravenously into murine breast cancer tumor-bearing mice for 2 h, conspicuous brightness in tumor regions was observed by CT imaging, with CT values significantly increased from 36.6 ± 11.1 before injection to 209.8 ± 40.1 after injection, demonstrating the feasibility of NaHoF_4_ NPs as CT contrast agents for tumor imaging.

Er, another heavy rare earth element with a K-edge at 57.5 keV, provides the necessary contrast for CT imaging and can be used for blood pool imaging 75. Gillies *et al.* synthesized an oleate-coated NaErF_4_ NPs as an excellent CT contrast agent *via* nanoprecipitation method for blood pool imaging *in vivo*, providing high contrast between the vessels and surrounding tissues due to their high contrast element loading (>100 mg/mL) and long circulation times (>1 h) (**Figure 4E**) 76. ​CT values of the heart and aorta increased by more than 250 HU compared to pre-injection when the contrast agent was injected intravenously into C57BL/6 male mice. Also, the heart chambers did not appear to change in contrast for up to 60 min.

Similarly, Yb with a higher atomic number (Z = 70) and K-edge energy (61 keV) than other Ln elements except for Lu, can be used as a high-performance CT contrast agent for *in vivo* angiography and lymph node mapping 77. Lu *et al.* developed PEGylated Yb-based contrast agents (NaYbF_4_ NPs) with negligible cytotoxicity and prolonged circulation time (>1 h)* in vivo* for CT imaging (**Figure 4F**) 78. The CT value of NaYbF_4_ NPs was 115 HU at 120 KVp, which was higher than most metallic contrast agents such as Au-based (65 HU) and Bi-based NPs (85 HU), since the K-edge of Yb was located within a higher energy region of the X-ray spectrum. After intravenous injection, a clear signal enhancement of the large vessels and lymph nodes was observed at 20 min post-injection (from 56.1 HU to 336.5 HU) and sustained for a minimum of 1 h.

Doping of Ln-based NPs can alter the longitudinal or transverse relaxation times and improve the conversion luminescence efficiency of activated ions because of their unique 4f electronic structure. Thus, Ln-based NPs are also used in MRI and optical imaging to remedy the deficiencies of CT imaging 79-82. Li *et al.* fabricated a polydopamine (PDA)-coated NaYF_4_:Nd^3+^@NaLuF_4_ nanocomposites using the reverse microemulsion approach for NIR-II optical imaging and CT imaging to provide accurate information on tumors (such as spatial position and anatomical details) (**Figure 4G**) 83. Due to the high X-ray attenuation coefficient of Lu (Z = 71), the slope of the CT values for the NaYF_4_:Nd^3+^@NaLuF_4_ nanocomposites was approximately 45.23 HU L/g, which was much higher than that for iodixanol (20.37 HU L/g). The luminescent NIR-II signal was captured upon irradiation by an 808 nm laser due to doping neodymium (Nd^3+^).

After intratumoral injection of NaYF_4_:Nd^3+^@NaLuF_4_ into HeLa tumor-bearing nude mice, the NIR-II and CT signals were observed at the tumor site, demonstrating its potential as a bimodal probe for tumor imaging. The combination of MRI and CT imaging provides more information on the tumor due to the high resolution of MRI in soft tissues 84. Yang *et al.* synthesized a Ce-doped NaCeF_4_:Gd,Tb scintillating nanoparticle (ScNPs) with uniform rod-like morphology* via* a high-temperature reaction method for CT and MR bimodal imaging (**Figure 4H**) 85. Because of the participation of Ce^3+^, Tb^3+^, and Gd^3+^ ions, the computed CT value slope was 38.152 HU L/g and the r_2_ relaxation was 39.89 mM^-1^ s^-1^, indicating the good performance of ScNPs in CT and *T*_2_-weighted MR imaging.

Almutairi *et al.* designed *β*-NaYb_0.2_/Er_0.8_F_4_@NaLuF_4_@NaGdF_4_ (Yb/Er@Lu@Gd) heteroepitaxial triple-layer core-shell-shell (CSS) NPs as a triple-modal contrast agent for photoluminescence (PL) imaging, MRI, and CT imaging (**Figure 4I**) 86. The triple-modal contrast agent had many advantages: (1) PL intensity was enhanced from completely quenched state to brightly emissive state at 808 nm irradiation, (2) the r_1_ relaxivity of MRI was enhanced by 5-fold from 11.4 to 52.9 mM^-1^ s^-1^ (per Gd^3+^) at 1.5 T, and (3) the CT value gain was 70 % higher than the iodinated contrast agents at the same mass concentration.

### Transition metal-based nanoparticles

#### Hafnium

**Figure 5:**
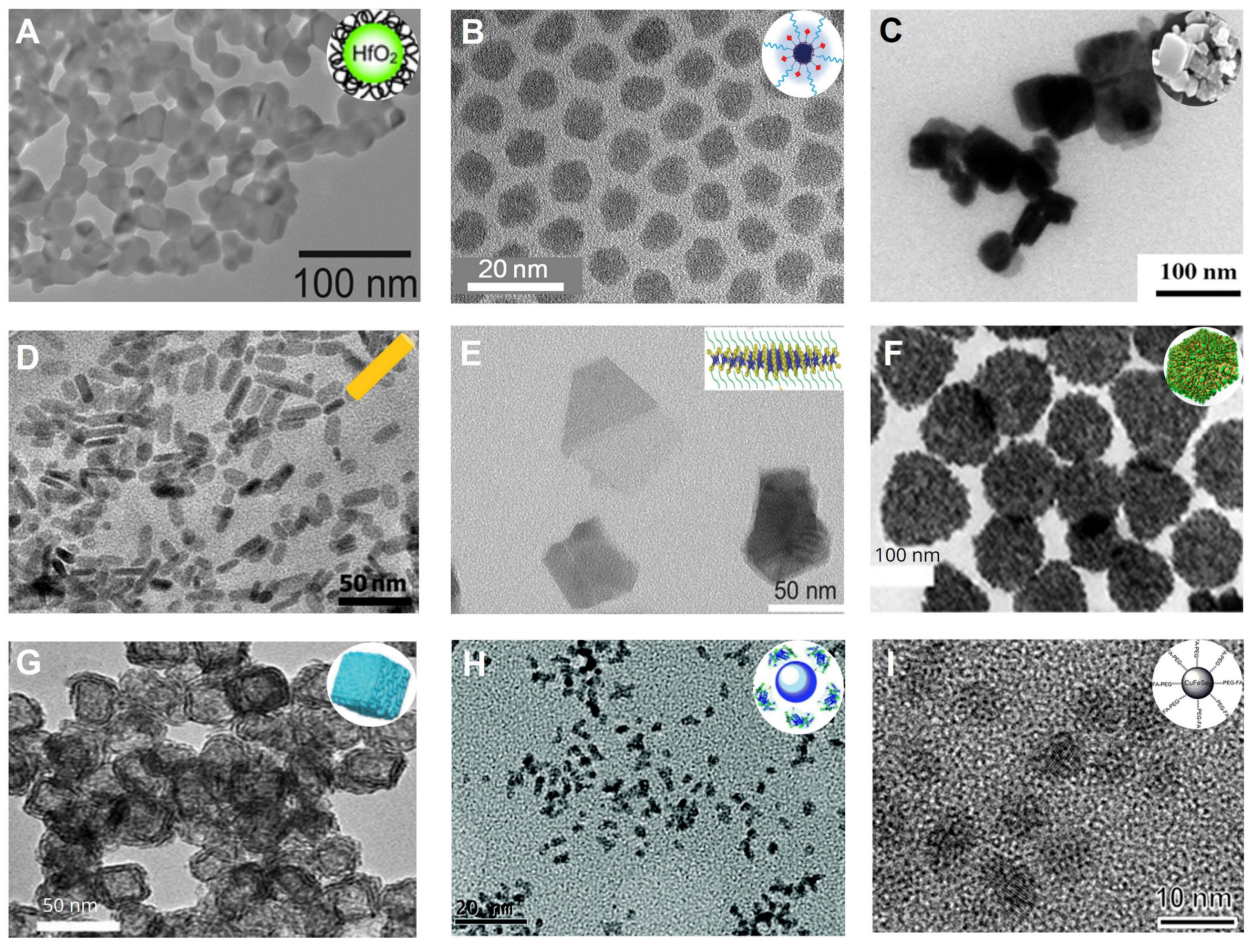
Transition metal-based NPs. TEM images of **(A)** PVP-HfO_2_ NPs, **(B)** PEG-TaO_x_ NPs, **(C)** PCL/PEG-coated WO_3_ NPs, **(D)** PVP-coated Rb_x_WO_3_ NRs, **(E)** PVPcapped ReS_2_ NSs, **(F)** IrNPs, **(G)** Pt Spirals, **(H)** Pt@BSA NPs, **(I)** CuFeSe_2_-PEG-FA NPs. **(A)** Adapted with permission from 89, Copyright 2016 The Royal Society of Chemistry. **(B)** Adapted with permission from 92, Copyright 2011 American Chemical Society. **(C)** Adapted with permission from 96, Copyright 2013 Elsevier. **(D)** Adapted with permission from 97, Copyright 2014 Wiley-VCH. **(E)** Adapted with permission from 99, Copyright 2018 Wiley-VCH. **(F)** Adapted with permission from 101, Copyright 2019 American Chemical Society. **(G)** Adapted with permission from 103, Copyright 2019 Wiley-VCH. **(H)** Adapted with permission from 104, Copyright 2017 Royal Society of Chemistry. **(I)** Adapted with permission from 105, Copyright 2021 Dove Press Ltd.

Hafnium (Hf) can be used as an excellent CT contrast agent as it has a high atomic number (Z = 72), and its K-side energy (65.4 keV) is close to the peak tube potential of clinical CT system (80-120 kVp) 87, 88. Roeder *et al*. produced roughly spherical and monodispersed PVP functionalized HfO_2_ NPs with an adjustable mean diameter of 7-31 nm *via* a sol-gel method (**Figure 5A**) 89. The X-ray attenuation of HfO_2_ NPs (11.1 HU/mM) at 0.5-50 mM concentration was intermediate between iohexol (6.8 HU/mM) and AuNPs (15.3 HU/mM). HfO_2_ and AuNPs exhibited significantly improved CT contrast compared to iohexol due to the Hf and Au K-shell absorption edges at 65.4 and 80.7 keV, respectively. Therefore, HfO_2_ can be used as a potential CT contrast agent to provide a better CT contrast effect than iohexol; it is also much cheaper than Au NPs (0.87 USD/g Hf *vs.* 64.06 USD/g Au).

#### Tantalum

Compared to other transition metals, tantalum (Ta) is a highly biocompatible metal that causes minimal adverse biological reactions in any redox form. Therefore, Ta compounds have been widely employed in clinical implants, artificial joints, stents, and vascular clips for about 50 years 90. In recent years, Ta has been used as a CT contrast agent due to its high atomic number (Z = 73) and K-edge energy (67 keV) in the uppermost energy region of the X-ray spectrum 91. Hyeon *et al.* prepared uniform-sized tantalum oxide (TaO_x_) NPs by a simple microemulsion method (**Figure 5B**) 92. The CT contrast enhancement of the TaO_x_ NPs (4.30 cm^2^/g, at 100 keV) was much higher than iodinated CT contrast agents. While TaO_x_ NPs smaller than 6 nm were cleared from the circulation within seconds after intravenous injection, the larger ones stabilized with PEG had long circulation times (over 3 h) and finally accumulated in the liver and spleen. Therefore, TaO_x_ NPs are potentially useful for angiography and RES-targeted CT imaging. Shi *et al.* developed biocompatible PEGylated tantalum sulfide (PEG-TaS_2_) nanosheets (NSs) by combinatorial grinding and sonication for CT imaging 93. At 24 h after intravenous administration, the CT values of tumor site increased from 21.3 HU to 44.6 HU. Furthermore, the CT signals in the heart at 2 h post-injection were much brighter than the pre-injection signals, indicating their longer circulation time.

#### Tungsten

Tungsten (W) is considered an appropriate candidate for CT imaging due to its high atomic number (Z = 74) and X-ray absorption coefficient (4.44 cm^2^/kg, at 100 keV) 94. However, the toxic nature of the W compound caused death in mice just seconds after intravenous administration 95. The most likely reason of this toxicity is the aggregation of particles in capillaries and arteries, resulting in embolism and subsequent clogging of blood vessels. To address these issues, Jakhmola *et al*. designed poly-ε-caprolactone (PCL) and PEG-modified tungsten oxide (WO_3_) NPs for *in vivo* CT imaging (**Figure 5C**) 96. The PCL/PEG-coated WO_3_ NPs showed good stability under physiological conditions and were non-toxic because the PCL layer inhibited particle agglomeration. The CT value of PCL/PEG-coated WO_3_ NPs was approximately 4-fold higher than the iodinated contrast agent (Fenestra VC). Zhao *et al.* reported PVP-coated rubidium tungsten bronze (Rb_x_WO_3_) NRs for CT imaging (**Figure 5D**) 97. The slope of CT values of PVP-coated Rb_x_WO_3_ NRs was approximately 38.66 HU L/g, much higher than that of Ultravist (13.25 HU L/g), indicating the good contrast efficacy of Rb_x_WO_3_ NRs for CT imaging. ​Immediately after the intratumoral injection, the CT signal at the tumor region was visible, demonstrating that RbxWO_3_ NRs can be employed as an effective contrast agent for *in vivo* CT imaging.

#### Rhenium

Rhenium (Re) possesses a high Z element (Z = 75) and strong Xray attenuation; hence, Re-based nanomaterials can also serve as an excellent CT contrast agent 98. Xu *et al.* prepared PVP-capped colloidal rhenium disulfide (ReS_2_) NSs with excellent physiological stability and high biocompatibility through a probe sonication-assisted liquid exfoliation method for *in vivo* CT imaging (**Figure 5E**) 99. The ReS_2_ NSs showed a superior CT enhancement effect over iopromide, as their Xray absorption coefficient (20 HU mL/mg) was higher than that of iopromide (15.9 HU mL/mg). After intratumoral injection, the CT values of the tumor region increased from 28.4 to 271.6 HU, indicating that PVPcapped ReS_2_ NSs were an excellent CT imaging contrast agent.

#### Iridium

Iridium (Ir), one of the least abundant noble metal elements with a high atomic number (Z = 77) and K-edge energy (71 keV), has attractive physical and chemical properties but has not been studied as a CT contrast agent due to the high price 100. To overcome the current limitations, Jang *et al.* prepared Ir-Ag-IrO_2_ nanoplates (IrNPs) *via* the hydrothermal galvanic replacement-tethered synthetic method for CT imaging (**Figure 5F**) 101. The CT value in the tumor region increased from 42.6 HU to 53.2 HU 24 h after intravenous administration, indicating the feasibility of CT imaging to monitor IrNPs accumulation in the tumor.

#### Platinum

Platinum (Pt), like gold, is another inert noble metal with strong X-ray attenuation and high atomic number (Z = 78). Therefore, Pt can potentially be used as a CT contrast agent for disease diagnosis 102. Zhang *et al.* designed unique PEG-modified hollow Pt spirals with a multilevel structure *via* a simple selective etching strategy (**Figure 5G**) 103. Due to its high K-edge energy (78.4 keV), Pt spirals had better contrast than a clinical iodinated CT contrast agent (Omnipaque) at the same concentration (5.39 *vs.* 4.76 HU/mM). A considerable enhancement of CT signals in the tumor region was detected (1.45-fold higher than before injection) 24 h after intravenous injection, showing that the Pt spirals were an excellent CT contrast agent for *in vivo* imaging. In addition, Jia *et al.* fabricated ultrasmall Pt@BSA NPs through a fast albumin-mediated one-pot synthesis method for CT imaging (**Figure 5H**) 104. These well-dispersed Pt@BSA NPs with a mean core size of 2.1 nm exhibited excellent colloidal stability, hemocompatibility, and biocompatibility. The CT value of Pt@BSA was approximately 2.4-fold higher than that of Ultravist at the same concentration. The contrast enhancement of the heart was observed (76 ± 4 HU) 5 minutes after intravenous injection of Pt@BSA NPs. However, the contrast enhancement of the heart was not observed 5 minutes after intravenous injection of Ultravist, indicating CT imaging capability of Pt@BSA NPs was superior to current clinical iodinated contrast agents.

#### Other transition metal-based NPs

It is generally believed that higher atomic numbers imply better X-ray attenuation coefficients and contrast enhancement. However, the transition metal-based NPs (such as Cu, Ag) with atomic numbers less than iodine can also be used as CT contrast agents by increasing local concentration, such as targeted aggregation. Shu *et al.* synthesized CuFeSe_2_-PEG-FA NPs with excellent targeting capability for CT imaging (**Figure 5I**) 105. Upon treatment with the CuFeSe_2_-PEG-FA NPs, the CT value (14.1 HU) of 4T1 cells was higher than that of treatment with CuFeSe_2_ NPs (1.2 HU) or Ultravist (8.2 HU), because folic acid (FA) targeted the overexpressed FA receptors in malignant tumors. Furthermore, the enrichment concentration of contrast agents *in vivo* could be enhanced by improving their biocompatibility 106. Wang *et al.* developed spherical, ultrasmall, and monodisperse HA-coated silver NPs (HA-Ag NPs) by the chemical reduction protocol for CT imaging 107. The HA-Ag NPs exhibited excellent long-term stability, low cytotoxicity, and enhanced CT contrast due to the HA-improved biocompatibility of HA-Ag NPs and facilitated the intratumor enrichment of the contrast agent.

Although commercial iodinated CT contrast agents have been developed from the first generation of ionic monomers to the third generation of non-ionic dimers, their inherent defects are still confusing, such as short blood pool circulation time and nonspecific biodistribution, requiring larger doses of contrast agents to reveal areas of interest. ​Consequently, AuNPs, with high contrast, easy control of size and surface modifications, controlled half-life, and targeted biodistribution, have attracted intense research in the last two decades. However, the high cost of gold limits its development and use in biomedicine. Bi-based NPs, with better X-ray attenuation properties and lower cost than AuNPs, have been widely used in the biomedical field as a suitable alternative, but encephalopathy and nephropathy caused by their long-term accumulation in the body remain challenging. Lanthanide ions such as Gd^3+^ have been used as commercial MRI contrast agents, indicating their promising potential for clinical applications. However, as CT contrast agents, lanthanide-based nanomaterials require a relatively high unit dose to enhance contrast, inevitably increasing the cost and toxicity risks. We tabulated the properties and characteristics of the representative CT contrast agents for clarity of presentation (**Table 2**).

## CT image-guided therapy

Current non-surgical therapeutic options for cancer mainly include chemotherapy and radiotherapy (RT), causing significant collateral damage 108. Although chemotherapeutic drugs kill cancer cells, they may cause severe harm to normal cells due to their non-selective effects. Radiation employed in RT may impact neighboring organs due to oxygen-free radicals produced during the radiation 109, 110. New nanoagents with diagnosis and treatment functions have been developed benefiting from molecular imaging and nanotechnology. The nanoagents are utilized in photothermal therapy (PTT), photodynamic therapy (PDT), gene therapy (GT), immunotherapy, gas therapy, sonodynamic therapy (SDT), starvation therapy *etc*. These emerging treatment modalities are expected to mitigate the side effects of traditional treatments and improve treatment efficiency. Here, we will focus on CT image-guided cancer treatment.

### Photothermal therapy

**Figure 6:**
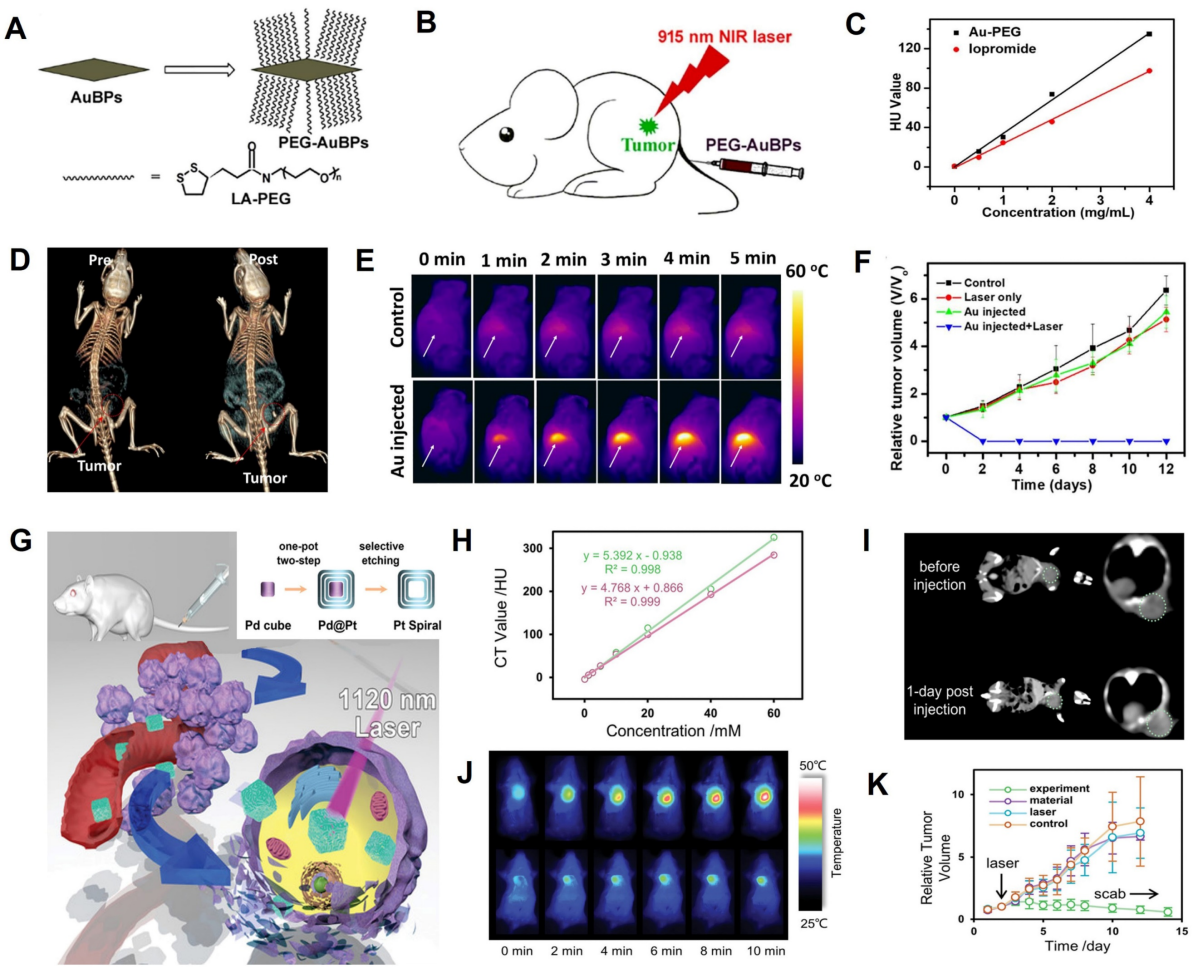
CT image-guided PTT. **(A)** Synthesis and modification of PEG-AuBPs. **(B)** Schematic illustration of AuBPs for CT image-guided PTT. **(C)** Concentration-dependent change in CT values of PEG-AuBPs and iopromide. **(D)** CT images of 4T1 tumor-bearing mice at pre- and post-intravenous injection. **(E)** Changes in thermal images of 4T1 tumor-bearing mice. **(F)** Relative tumor volume after various treatments. **(G)** Schematic diagram of the synthesis and treatment of Pt Spirals. **(H)** CT values of Pt Spirals and Omnipaque at different concentrations. **(I)** CT images of tumor-bearing mice at pre- and post-intravenous injection of Pt Spirals. **(J)** Thermal images of tumor-bearing mice. **(K)** Relative tumor volume in different groups.** (A-F)** Adapted with permission from 119, Copyright 2019 Elsevier. **(G-K)** Adapted with permission from 103, Copyright 2019 Wiley-VCH.

PTT is a promising cancer therapy method that uses NIR light to excite photothermal agents (PTAs) delivered to the tumor region 111, 112. PTAs absorb and convert light energy to heat, resulting in local hyperthermia to kill cancer cells 113-116. AuNPs, including hollow spherical structures 117, nanorods 118, bipyramids 119, and nanostars 120, are excellent CT contrast agents and PTAs due to their high X-ray attenuation coefficient and superior photothermal conversion efficiency (PCE) 121. Zhang *et al.* designed PEG-functionalized monodisperse gold bipyramids (AuBPs) with controlled aspect ratio and tunable surface plasmon resonance (SPR) property, which can tune the absorption wavelength to NIR region and significantly enhance the light absorption cross-section for CT image-guided PTT (**Figure 6A-B**) 119. The CT value increased linearly with the increasing concentration of PEG-AuBPs and was higher than that of iopromide at the same concentration (**Figure 6C**). After intravenous administration of PEG-AuBPs to 4T1 tumor-bearing mice, the contrast at the tumor site increased significantly over time and reached a maximum at 24 h post-injection, indicating significant enrichment of PEG-AuBPs at the tumor site (**Figure 6D**). Given the CT imaging result, PTT was carried out at 24 h post-injection and the temperature of the tumor site increased from 35 °C to 52 °C after 5 min of 915 nm laser irradiation (**Figure 6E**), generating hyperthermia to kill tumor cells effectively (**Figure 6F**).

However, limited by low maximum permissible exposure (MPE, 0.33 W cm^-2^) of the first near-infrared bio-window (NIR-I, 700-1000 nm) for skin 122, the efficacy of PTT was poor 123. PTT triggered by the second near-infrared bio-window (NIR-II, 1000-1350 nm), generated widespread concern due to its deeper penetration depth of biological tissues, lower photon scattering, and higher MPE (1.0 W cm^-2^) 124. Wang *et al*. synthesized hollow Pt nanoframes (“Pt spirals”) by a simple selective etching strategy, which exhibited high PCE (52.5%) and strong molar extinction coefficients (MEC, 228.7 m^2^ mol^-1^) in NIR-II window (**Figure 6G**) 103. Pt nanostructures also had a high K-edge value (78.4 keV), endowing them with great potential for CT image-guided PTT. Also, Pt spirals exhibited higher contrast efficacy than the iodine-containing agent (Omnipaque) at equivalent concentrations (**Figure 6H**); 24 h after intravenous injection of Pt spirals, the CT value of the tumor site increased from 37.5 HU to 58.2 HU (**Figure 6I**), indicating the maximum enrichment. Therefore, 24 h was selected as the starting time point of treatment. Based on the location information provided by CT imaging, the tumor region of the mouse was irradiated with an 1120 nm laser at 24 h post-injection. The temperature in the tumor region reached an average value of 49.9 °C in the experimental group after 10 min of irradiation, which was significantly higher than (41.9 °C) in the control group (**Figure 6J**), and the tumor completely ablated at 10 days post-treatment (**Figure 6K**). These results illustrated that CT image-guided PTT might hold great potential for future biomedical applications.

### Photodynamic therapy

PDT takes advantage of the cytotoxicity of ROS generated by the reaction of photosensitizers (PSs) and oxygen (O_2_) in light irradiation to kill tumor cells 125. However, hypoxic TME and low light penetration depth limit the application of PDT in biomedicine. Therefore, multifunctional nanoagents with CT imaging, NIR absorption, and catalytic O_2_ generation capabilities are expected to address the PDT disadvantage.

**Figure 7:**
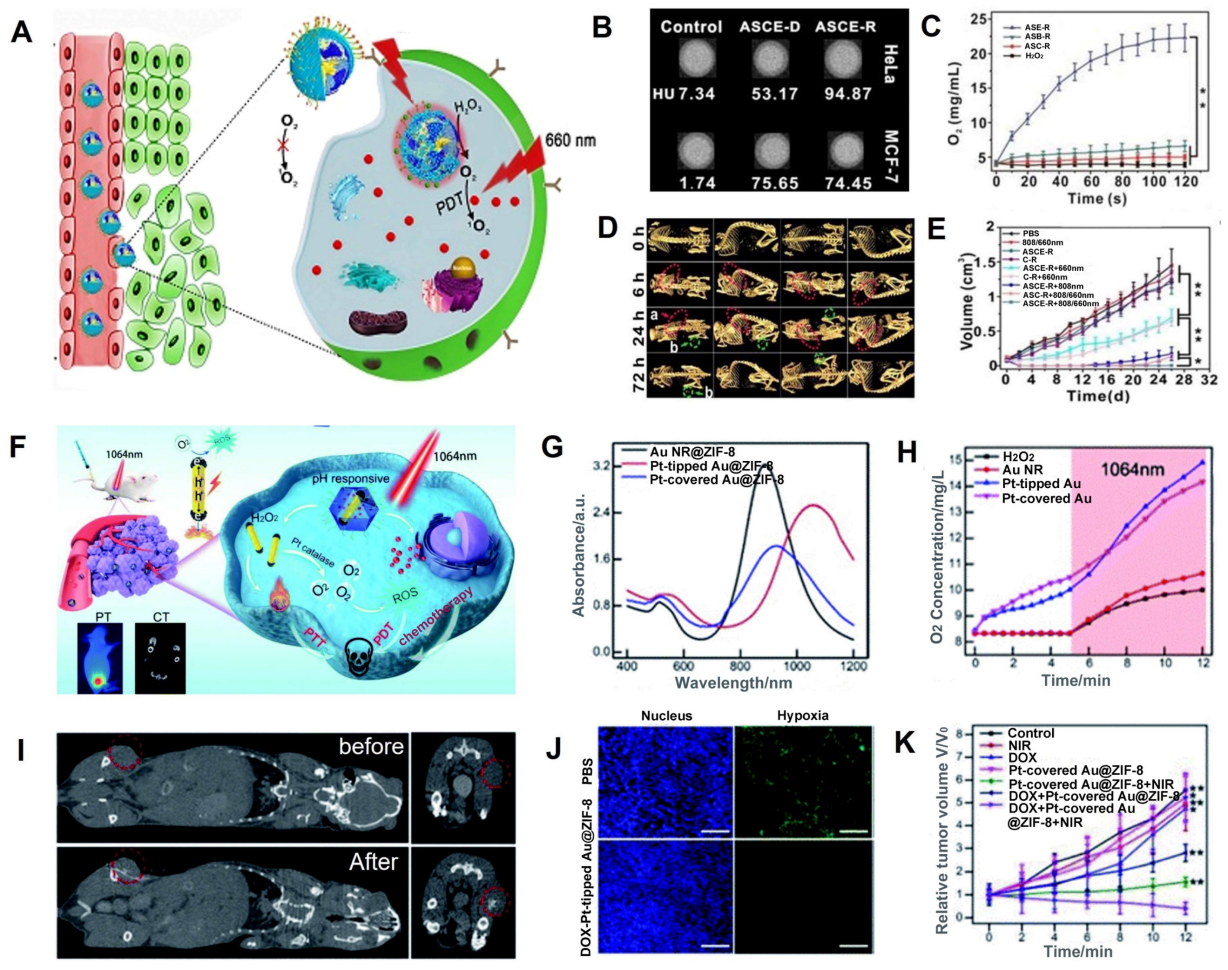
CT image-guided PDT. **(A)** Schematic illustration of CT image-guided PDT. **(B)** CT signal intensity of the nanomaterial. **(C)** O_2_ production curve of different probes. **(D)** *In vivo* CT imaging. **(E)** Tumor volume after different treatments. **(F)** Schematic illustration of the CT image-guided PDT. **(G)** UV-vis-NIR absorption spectra of Pt-tipped Au@ZIF-8. **(H)** O_2_ generation under 1064 nm laser irradiation. **(I)** CT images of tumor-bearing mice before and after intratumoral injection. **(J)** HIF-1a staining of 4T1 tumor in different treatment groups. **(K)** Relative tumor volume after different treatments. **(A-E)** Adapted with permission from 128, Copyright 2019 Ivyspring International Publisher. **(F-K)** Adapted with permission from 130, Copyright 2021 The Royal Society of Chemistry.

Hypoxia in the tumor may be alleviated by catalyzing the production of O_2_ from H_2_O_2_, which is overexpressed in cancer cells 126, 127. Zhao *et al.* designed c(RGDyK)-targeting and catalase-modified Ce6-loaded AuNSs (ASCE-R) for CT image-guided PDT (**Figure 7A**) 128. Because of the high expression of α_v_β_3_ receptor in HeLa cells, the CT values of HeLa cells incubated with ASCE-R exhibited higher CT values (94.85 HU) compared with MCF-7 cells (low expression of the α_v_β_3_ receptor) incubated with ASCE-R (74.45 HU), indicating that ASCE-R presented outstanding tumor-targeting, CT imaging capability (**Figure 7B**). Meanwhile, the ASCE-R showed an excellent O_2_ production rate (23 mg/mL, **Figure 7C**), confirming that catalase in the probe could catalyze H_2_O_2_ to O_2_. The CT signal at the tumor site was observed 24 h after injection of the ASCE-R probe and peaked at 72 h, demonstrating the excellent intratumoral retention capability of ASCE-R (**Figure 7D**). At this time point, the tumor site was irradiated with the NIR laser at 660 nm, and the tumor perished 26 d post-irradiation, demonstrating the excellent CT image-guided PDT effect achieved by ASCE-R as a versatile nanoagent (**Figure 7E**).

​Furthermore, light in the NIR-II window affords greater penetration depth and lower photo-toxicity to normal tissues than light in the visible and NIR-I windows 129. Xu *et al.* fabricated isotropically plasmonic bimetal Pt-tipped AuNRs with zeolitic imidazolate framework-8 (Pt-tipped Au@ZIF-8) for CT image-guided PDT, which presented efficient plasmon-induced electron-hole spatial separation with 1064 nm laser irradiation and excellent CT imaging performance (**Figure 7F-G**) 130. Meanwhile, nano-Pt with catalase-like activity could catalyze H_2_O_2_ overexpressed in TME to generate O_2_ and relieve tumor hypoxia (**Figure 7H**). After injection, a significantly enhanced CT signal was observed at the tumor site. Thus, CT imaging could be used to determine the region of NIR-II laser irradiation and monitor the treatment effect (**Figure 7I**). Tumor tissue showed significantly weak hypoxia green fluorescence 24 h after injection of Pt-tipped Au@ZIF-8 by immunofluorescence staining assay with hypoxia-inducible factor (HIF)-1a (**Figure 7J**), demonstrating that Pt-tipped Au@ZIF-8 could efficiently alleviate tumor hypoxia. Also, tumor volume was significantly reduced in the Pt-tipped Au@ZIF-8 group after 12 days of treatment with 1064 nm laser irradiation guided by CT imaging (**Figure 7K**).

### Chemotherapy

Conventional chemotherapy drugs have several drawbacks, including low selectivity, high toxicity, and insufficient efficacy 131. The CT-guided NP delivery systems can overcome the limitations of traditional chemotherapy 132 by targeting the release of chemotherapy drugs to the tumor region, and have several advantages, including (1) controlled release in a specific site triggered by specific stimuli to reduce damage to normal tissues 133, (2) targeted drug release to cancer cells to reduce side effects and increase the concentration of the drug in the target region 134, and (3) combined contrast agents to improve the efficiency of diagnosis and treatment 135.

**Figure 8:**
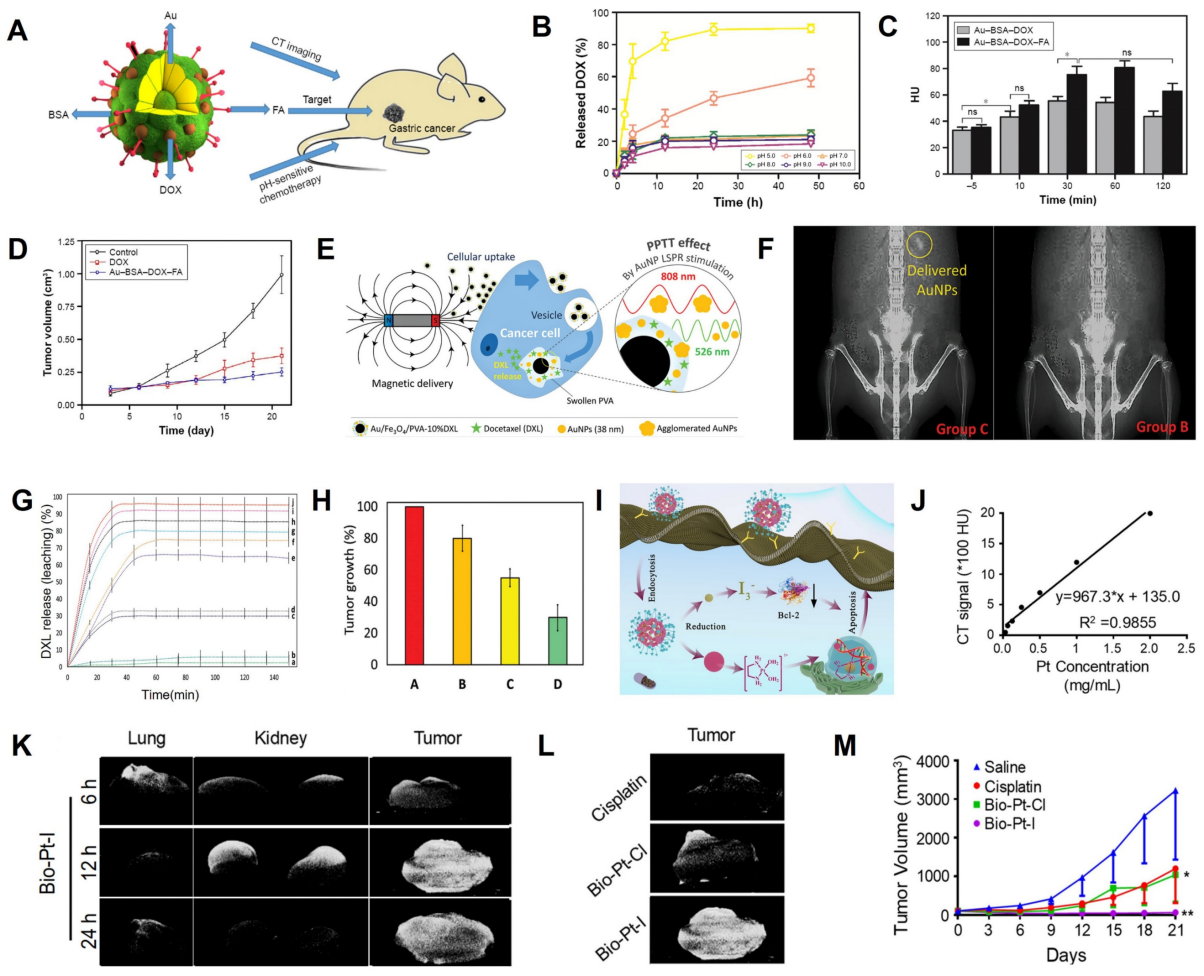
CT image-guided chemotherapy. **(A)** Schematic illustration of Au-BSA-DOX-FA nanocomposite for CT image-guided chemotherapy. **(B)** Release of DOX at different pH values. **(C)** CT values of gastric cancer tissues. **(D)** Tumor volumes. **(E)** ​Schematics of magnetically-delivered and released drugs, and **(F)** Corresponding CT images. **(G)** Release profiles of DXL from Au/Fe_3_O_4_/PVA-10%DXL. **(H)** Tumor growth at 14 days post-treatment. **(I)** Antitumor mechanism of I-Pt-Bio NPs. **(J)** CT values at various concentration of Bio-Pt-I NPs. **(K)** CT images of major organs of mice at different times. **(L)** CT images of mice after treatment with Bio-Pt-Cl and Bio-Pt-I for 12 h. **(M)** Tumor growth curves after different treatments. **(A-D)** Adapted with permission from 136, Copyright 2017 Dove Press Ltd.** (E-H)** Adapted with permission from 137, Copyright 2020 Wiley-VCH. **(I-M)** Adapted with permission from 140, Copyright 2022 American Chemical Society.

Wang *et al.* prepared a drug delivery carrier (Au-BSA-DOX-FA NPs) consisting of BSA-modified Au NPs and anhydride-doxorubicin (cis-DOX) and FA for CT image-guided targeted chemotherapy in cancer tissues overexpressing folate receptor (FR) (**Figure 8A**) 136. Quantification of DOX in different pH media indicated that the aconityl linker between DOX and Au-BSA NPs was readily broken and released about 90% DOX at pH 5.0 (**Figure 8B**), indicating excellent pH-responsive drug release capability of Au-BSA-DOX-FA NPs. After intravenous administration, the CT values of the tumor site in the Au-BSA-DOX-FA NP-targeted group were markedly higher than the non-targeted group at 30 min post-injection (**Figure 8C**), indicating that the TME-responsive drug delivery system could achieve targeted drug delivery. In addition, the tumor volume at 20 days after intravenous administration of Au-BSA-DOX-FA was significantly reduced compared with the control group (0.25 *vs*. 0.88 cm^3^, **Figure 8D**).

Magnetic nanocomposites, such as Fe_3_O_4_ NPs, loaded with chemotherapy drugs can also be released into target tumor tissues *via* magnetic orientation and CT localization. Kitaev *et al.* fabricated a polyvinyl alcohol (PVA)-encapsulated DXL (docetaxel, a chemotherapeutic drug)-loaded dual (temperature and pH)-responsive magnetically directed plasmonic nanocomposite (Au/Fe_3_O_4_/PVA-10%DXL) for CT image-guided chemotherapy 137. ​After 12 h of application of a 1 Tesla magnetic field to the tumor-bearing mouse, the Au/Fe_3_O_4_/PVA-10%DXL nanocomposite was navigated to the cancer site, as confirmed by CT scans (**Figure 8E-F**). At this time point, the high temperature produced by 808 nm NIR irradiation and acidic TME controlled DXL release. Thus, Au/Fe_3_O_4_/PVA-10% DXL exhibited efficient selective release (≈ [96 ± 3]% of DXL) and the tumor inhibition effect ([70 ± 6.3]%) (**Figure 8G-H**).

Pt can be used as a CT contrast agent and, due to its inherent antitumor effect, can also be used for synthesizing chemotherapy drugs, such as Cisplatin and Carboplatin 138. However, high concentrations of Pt must be maintained to obtain satisfactory CT imaging results, raising the possibility of serious side effects 139. Recently, Huang *et al.* synthesized reductant-sensitive bio-Pt-I NPs consisting of iodine-conjugated Pt (IV) compounds and biotin as a theranostic nanomedicine for CT image-guided treatment (**Figure 8I**) 140. The bio-Pt-I NPs with high Pt (25.36%) and I (32.99%) contents could be used for CT imaging and real-time dynamic distribution monitoring of nanomedicines. CT signal intensity increased as a function of bio-Pt-I NP concentration (**Figure 8J**). After intravenous administration of bio-Pt-I NPs, the CT signal at the tumor site was significantly enhanced at 6 h, peaked at 12 h, and remained until 24 h post-injection, enabling visualization of the enrichment of bio-Pt-I NPs in tumors (**Figure 8K**). In addition, the bio-Pt-I exhibited a stronger CT imaging signal (1680 HU) than bio-Pt-Cl (1126 HU) at the same concentration due to the iodine element (**Figure 8L**). Furthermore, Pt (IV) in the bio-Pt-I NPs could be reduced to Pt (II) by GSH overexpressed in tumor cells, leading to a controlled release of active Pt (II) segments to improve antitumor outcomes (**Figure 8M**).

### ​Radiotherapy

Radiotherapy is one of the most effective treatment options for cancer, but radioresistance is a significant challenge 141-143. Therefore, it is imperative to develop nanoagents with radio-sensitization to improve the RT effect.

**Figure 9:**
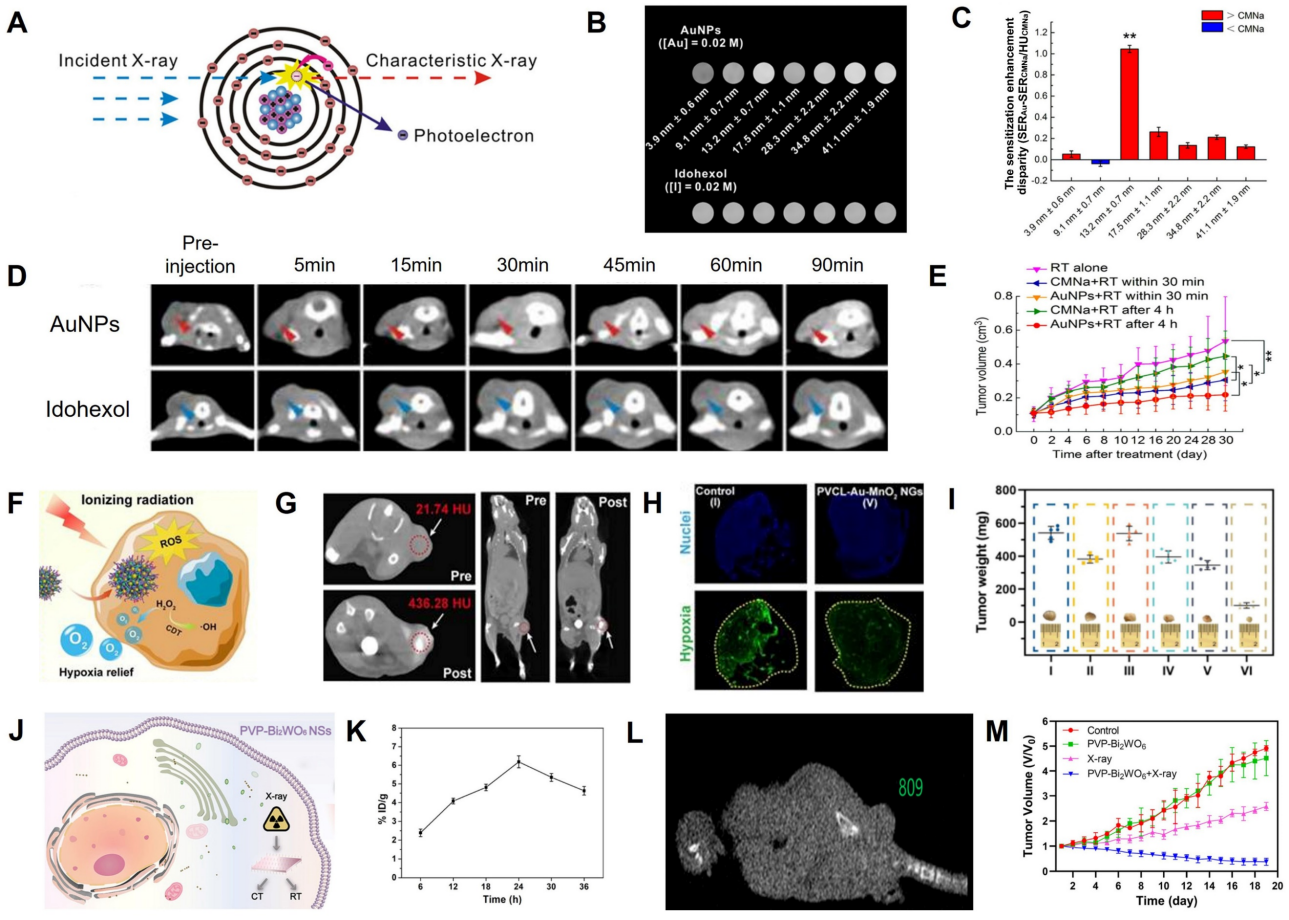
CT image-guided radiotherapy. **(A)** Proposed mechanism of the photoelectric effect. **(B)** CT signal intensities of different sizes of AuNPs and Iohexol at the same concentration. **(C)** Sensitization enhancement disparity between AuNPs and CMNa for various particle sizes under 6 Gy radiation. **(D)** *In vivo* CT images after intravenous administration of AuNPs and Iohexol. **(E)** Tumor growth inhibition curves after various treatments. **(F)** Schematic diagram of PVCL-Au-MnO_2_ NGs for radio-sensitization. **(G)** *In vivo* CT images of mice before and at 24 h post intravenous injection. **(H)** Hypoxia-positive immunofluorescence images. **(I)** Tumor weight after different treatments. **(J)** Schematic diagram of PVP-Bi_2_WO_6_ NSs for CT image-guided RT. **(K)** Aggregate amount and **(L)** CT values at 24 h post-intravenous injection of PVP-Bi_2_WO_6_ NS. **(M)** Tumor volume after various treatments. **(A-E)** Adapted with permission from 15, Copyright 2016 American Chemical Society. **(F-I)** Adapted with permission from 151, Copyright 2022 Ivyspring International Publisher. **(J-M)** Adapted with permission from 153, Copyright 2022 American Chemical Society.

The surface of AuNPs produces secondary electrons or Auger electrons *via* the photoelectric effect when it absorbs low and medium-energy X-ray (<250 keV), improving the local RT effect and reducing the damage to normal tissues due to the short range of the secondary electrons (**Figure 9A**) 144-146. Chang *et al.* synthesized PEGylated ∼13 nm AuNPs for CT image-guided RT because of their superior CT contrast and radio-sensitization properties 15. The CT value of PEGylated AuNPs was 1.2 times that of iohexol (**Figure 9B**) and their sensitization enhancement ratio (SER) was twice that of CMNa (a clinical radiosensitizer) (**Figure 9C**). Also, the CT signal at tumor region was 1.8-fold higher than iohexol 90 min after intravenous administration of the PEGylated AuNPs (**Figure 9D**). ​When the tumor was exposed to 6 Gy of X-ray irradiation at 90 min post-injection, the growth of the tumor was dramatically inhibited with a 61.26% increase in radio-sensitization compared to RT alone (**Figure 9E**).

Cancer cells are three times more radioresistant in hypoxia than in aerobic conditions, which has been attributed to the mechanism by which DNA damage by ionizing radiation can be easily repaired in the absence of O_2_ 147. MnO_2_ with a catalase-mimic catalytic activity can catalyze overexpressed H_2_O_2_ in tumors to generate O_2_, thus relieving tumor hypoxia 148-150. Shi *et al.* synthesized intelligent Au/MnO_2_-coloaded poly(N-vinylcaprolactam) (PVCL-Au-MnO_2_) nanogels (NGs) for CT image-guided RT; PVCL-Au-MnO_2_ NGs with a catalase-mimic catalytic activity could convert H_2_O_2_ in tumors to generate O_2_ to improve the radio-sensitization effect (**Figure 9F**) 151. The CT value increased linearly as a function of the PVCL-Au-MnO_2_ NG concentration. A significant CT signal enhancement at the tumor site (the CT value increased from 21.7 ± 1.6 HU to 436.3 ± 3.8 HU) was observed 24 h after intravenous injection (**Figure 9G**), indicating that PVCL-Au-MnO_2_ NGs enabled the efficient accumulation of O_2_ in tumor regions. ​Besides, immunofluorescence images and intensity analysis showed that the percentage of hypoxic tumor areas decreased from 67.6% at pre-injection to 25.3% at 24 h post-injection (**Figure 9H**), confirming that the PVCL-Au-MnO_2_ NGs could relieve tumor hypoxia. Therefore, CT imaging results were used to determine the location and start time of X-ray irradiation. ​When the tumor was exposed to 4 Gy of X-ray irradiation at 24 h post-injection, the growth of the tumor was significantly inhibited with an SER of ~1.49 at 20 days post-irradiation (**Figure 9I**), indicating that the PVCL-Au-MnO_2_ NGs possessed an excellent CT-guided radio-sensitization effect.

The radiosensitization effect of BiNPs is approximately 1.25-fold higher than AuNPs 152. Li *et al.* prepared PVP-modified Bi_2_WO_6_ NSs by the hydrothermal synthesis method for CT image-guided RT (**Figure 9J**) 153. The CT values and the accumulation rate at the tumor site was 809 HU and 6.19%, respectively, 24 h after intravenous injection of PVP-Bi_2_WO_6_ NSs (**Figure 9K-L**); therefore, this time point was identified as the point of initiation of treatment. The SER was calculated to be ~2.65 after 18 days of RT, and the tumors in mice were completely inhibited (**Figure 9M**).

### Immunotherapy

**Figure 10:**
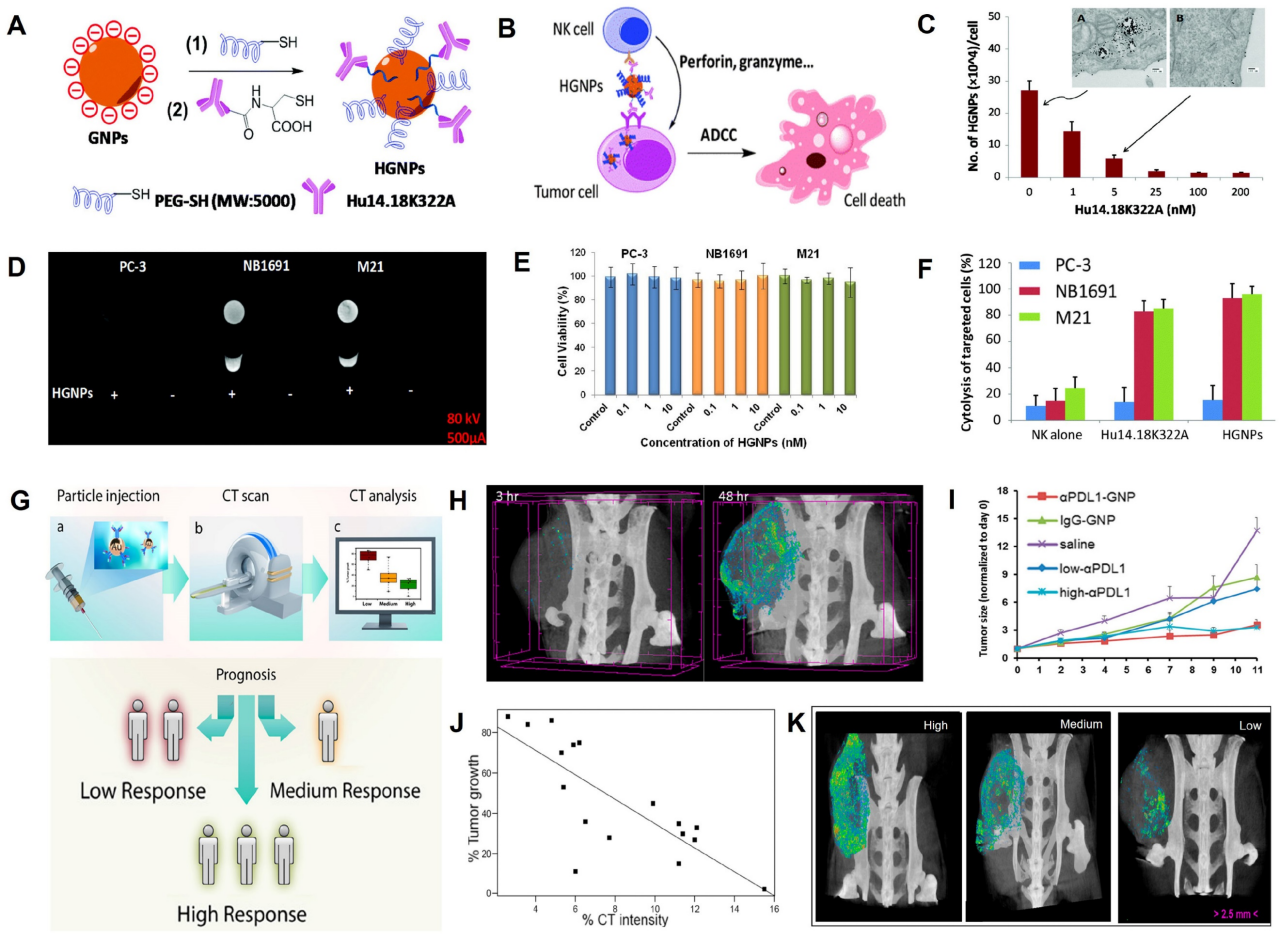
CT image-guided immunotherapy. **(A)** Synthesis of HGNPs. **(B)** Schematic illustration of HGNP-mediated immunotherapy. **(C)** Internalization of HGNPs in M21 cells. **(D)** CT images of PC-3, NB1691, and M21 cell pellets. **(E)** Cytotoxicity of HGNPs to the PC-3, NB1691, and M21 without NK cells. **(F)** Cytotoxicity of HGNPs to the PC-3, NB1691, and M21 with NK cells. **(G)** Schematic illustration of immune reactivity prediction *via* CT imaging. **(H)** CT signals in tumor sites of mice at different times. **(I)** Tumor growth rate in different treatment groups. **(J)** Correlation between CT signal intensity and therapeutic outcomes. **(K)** CT image-guided stratification. **(A-F)** Adapted with permission from 157, Copyright 2016 The Royal Society of Chemistry. **(G-K)** Adapted with permission from 163, Copyright 2017 American Chemical Society.

Immunotherapy often uses a combination of antigens and antibodies to help the host recognize the tumor and stimulate the immune system to activate immune cells to kill the tumor 154, 155. However, free antigens are easily inactivated by enzymes in blood circulation 155, 156. Therefore, nanomaterials with imaging capabilities are used as carriers for the targeted transport of these antigens, enabling antigen protection and real-time efficacy monitoring. Yan *et al.* designed a nanotracer (named HGNPs) consisting of AuNPs and hu14.18K322A (a tumor-targeting anti-GD2 antibody) for attacking and killing specifically cancer cells through antibody-dependent natural killer (NK) cell-mediated cytotoxicity (ADCC) and realizing CT image-guided immunotherapy (**Figure 10A-B**) 157. After incubation with HGNPs for 12 h, the CT signal intensity of neuroblastoma NB1691 and melanoma M21 cells (GD2-positive cells) was 5.27- and 7.66-fold, respectively, of that before incubation with HGNPs. While GD2-negative PC-3 cells remained essentially unchanged before and after incubation (**Figure 10C-D**), these results demonstrated that HGNPs had excellent cell-targeting capabilities. When combined with NK cells, HGNPs induced significant ADCC and killed almost 100% of the GD2-positive target, while there was no appreciable therapeutic effect on GD2-negative PC-3 cells, indicating that HGNPs had excellent targeted immunotherapy capabilities (**Figure 10E-F**).

Only a small number of patients can benefit from immunotherapy due to the lack of effective stratification of patients 158. Immune checkpoints overexpressed on tumor cells, such as programmed death ligand 1 (PDL1), inhibit the antitumor immunoreaction 159-162. However, immune checkpoint blockade (ICB) therapy has a limited success rate of no more than 30%; therefore, early identification of subjects who benefit from ICB therapy through CT imaging techniques is crucial to cancer treatment decisions. Popovtzer *et al.* designed programmed death ligand 1 antibody (αPDL1)-modified AuNP nanoagents (αPDL1-AuNPs) with CT imaging and therapy functions for achieving the integration of hierarchical diagnosis and treatment (**Figure 10G**) 163. The PDL1 expression dramatically reduced from 86.6% to 23.9% when MC38 colon carcinoma cells were incubated with αPDL1-AuNPs, demonstrating that the immune checkpoint ligand on the cell surface could bind with αPDL1-AuNPs and facilitate T cell activation. Moreover, αPDL1-AuNPs, as a CT contrast agent, were able to track the *in vivo* distribution and evaluate the treatment effect. After αPDL1-AuNPs were intravenously injected into MC38 colon carcinoma cell-bearing mice, the maximum accumulation of the nanoprobe occurred at 48 h post-injection (**Figure 10H**), and the tumor growth rate was significantly inhibited (**Figure 10I**). Subsequently, the early prediction of therapeutic response was performed by intravenously injecting αPDL1-AuNPs into 20 tumor-bearing mice, and the tumor size was measured at 48 h post-injection through CT scan and 8 days post-injection *via* calipers. The results showed that the quantitative CT signal at 48 h post-injection and tumor size at 8 days post-injection were linearly correlated (R^2^ = 0.6162, **Figure 10J**), demonstrating that the combination of αPDL1-AuNPs and CT imaging could predict therapeutic response as early as 48 h after treatment (**Figure 10K**).

### Starvation therapy

As an emergent cancer treatment strategy, starvation therapy could effectively inhibit tumor growth by cutting off the nutrient (glucose) supply of the tumor 164-166. Glucose oxidase (GOD) is a type of oxidoreductase that converts glucose, O_2,_ and water into gluconic acid and H_2_O_2_ 167. However, this reaction highly depends on the on-site O_2_ content, and alleviating hypoxia at the tumor site is a prerequisite for starvation therapy in treating tumors 168.

**Figure 11:**
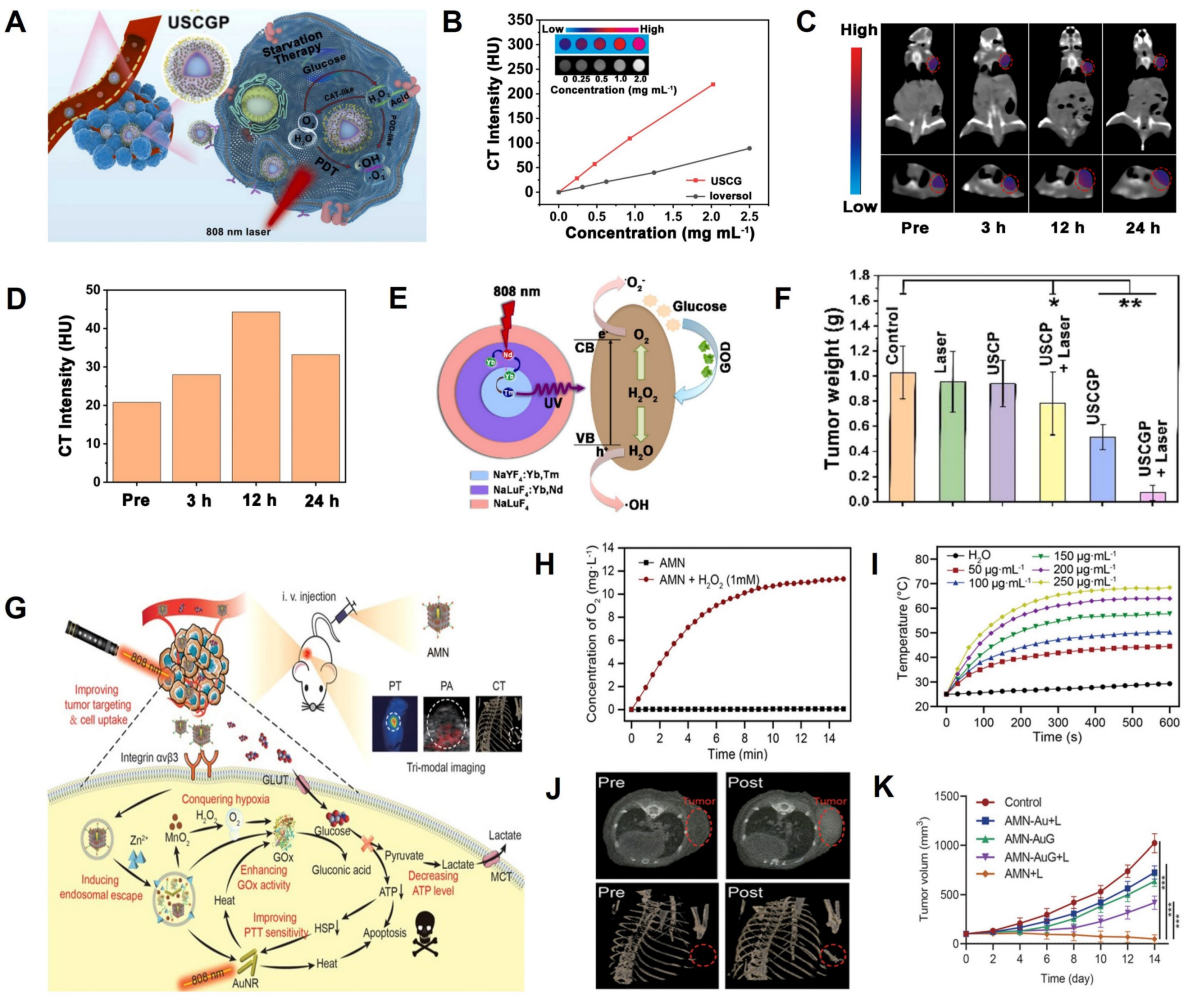
CT image-guided starvation therapy. **(A)** Schematic illustration of USCGP biocatalyst for starvation therapy. **(B)** Concentration-dependent CT signals of ioversol and USC. **(C)** CT contrast of 4T1 tumor-bearing mouse. **(D)** CT signal intensities of tumor area at different time points. **(E)** Photocatalysis mechanism of USCG. **(F)** Tumor weight after 14 days of treatment. **(G)** Mechanism of AMN for glucose metabolism. **(H)** Time-dependent AMN-catalyzed O_2_ generation. **(I)** Photothermal effect of AMN. **(J)** CT images of tumor-bearing mice pre- and post-injection of AMN. **(K)** Tumor volume changes. **(A-F)** Adapted with permission from 169, Copyright 2022 Elsevier.** (G-K)** Adapted with permission from 170, Copyright 2021 Wiley-VCH.

Zhang *et al.* developed a GOD-loaded catalase (CAT)-like biocatalyst (PEGylated UCNPs@mSiO_2_@CeO_2_-GOD, USCGP) with strong X-ray attenuation capability and O_2_ self-generation function for CT image-guided synergistic starvation therapy and PDT (**Figure 11A**) 169. The CT value gradually increased with the increasing concentration of USCGP and was 3-fold higher than ioversol at the same concentration, indicating the superior CT imaging capability of USCGP (**Figure 11B**). After intravenous injection of USCGP, the CT values at the tumor site increased gradually from 18.8 HU (pre-injection) to 48.3 HU (12 h post-injection), indicating that USCGP could effectively accumulate at the tumor site (**Figure 11C-D**). As shown in **Figure 11E**, when USCGP was internalized into tumor cells, intratumoral overexpressed H_2_O_2_ was decomposed into O_2_ by catalysis of USCGP with CAT-like activity. Catalyzed by GOD, the resulting O_2_ reacted with endogenous glucose to generate H_2_O_2_, and glucose was consumed for starvation therapy. In addition, highly toxic •OH was produced under NIR laser illumination due to the involvement of CeO_2_ with peroxidase (POD)-like activity and H_2_O_2_, further killing cancer cells through PDT. After intravenous administration, the tumor was effectively inhibited at day 14 post-treatment (**Figure 11F**), indicating USCGP is a promising theranostic agent for CT image-guided synergistic therapy.

Ning *et al.* developed an adenovirus-mimicking nanomachine (AMN) consisting of AuNRs, GOD, BSA-MnO_2_, and RGD peptide for image-guided synergistic starvation therapy and PTT (**Figure 11G**) 170. AMN possessed adenovirus-like function, excellent cancer cell uptake, and tumor targeting capability. AMN also facilitated glucose oxidase-triggered glucose oxidation *via in situ* oxygenation (**Figure 11H**) and photothermal effect (**Figure 11I**), which significantly inhibited the expression of heat shock proteins (HSPs) and, in turn, increased photothermal efficacy, leading to synergistic antitumor effects. After intravenous administration, the CT signal at the tumor site was detected at 4 h post-injection and reached a plateau at 12 h post-injection, indicating it to be the optimal timing (**Figure 11J**). ​Thus, the superior CT imaging capabilities of AMN enabled differentiation between tumors and normal tissues. Furthermore, AMN could selectively target the tumor region and effectively eliminate the tumor after intravenous administration (**Figure 11K**), demonstrating that the virus-like nanotheranostic platform could potentially promote the development of complementary modalities for cancer therapy.

### Gas therapy

**Figure 12:**
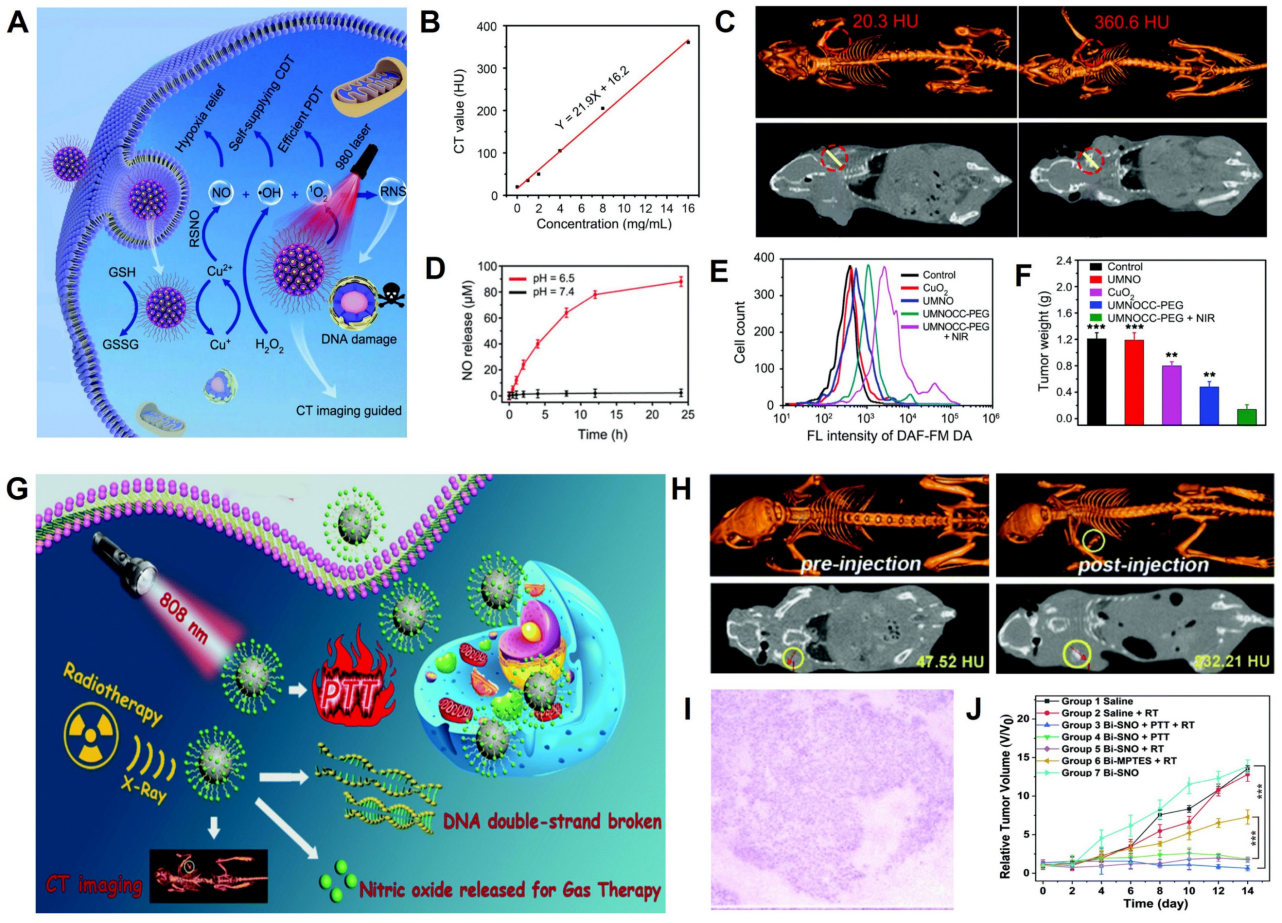
CT image-guided gas therapy. **(A)** Proposed mechanism of Cu^2+^-initiated multimodality therapy. **(B)** ​CT values as a function of concentration. **(C)** CT contrast of tumor-bearing mice at pre- and post-injection. **(D)** NO generation from UMNOCC-PEG under different pH values. **(E)** Intracellular NO by flow cytometry analysis. **(F)** Tumor weights of mice at 20-day post-treatment. **(G)** Schematic illustration of the Bi-SNO NPs for CT image-guided gas therapy. **(H)** CT contrast of tumor-bearing mice at pre- and post-injection. **(I)** H&E-stained image of tumor tissues. **(J)** Tumor volumes of mice in different treatment groups.** (A-E)** Adapted with permission from 174, Copyright 2020 The Royal Society of Chemistry. **(F-I)** Adapted with permission from 175, Copyright 2020 The Royal Society of Chemistry.

As a “green” cancer therapy approach, gas therapy (*e.g.*, NO, H_2_S, CO, H_2_, SO_2_, and CO_2_) has attracted increasing attention due to its low toxicity, minimal side effects, and low drug resistance 171. Nitric oxide (NO) is a vasodilatation factor that can relieve the hypoxia status of tumor cells and enhance their sensitivity to RT 172. The high concentration of NO can also induce apoptosis of cancer cells by producing oxidative and nitrative stress, inhibiting DNA repair and cellular respiration, and enhancing inflammatory reactions 173. Liu *et al.* developed a Cu^2+^-initiated NO-releasing nanoagents (UMNOCC-PEG) consisting of dendritic porous SiO_2_, UCNPs, SNO, Ce6, and CuO_2_ for CT image-guided synergistic gas, chemodynamic, and photodynamic therapy (**Figure 12A**) 174. The CT value increased linearly with increasing concentration of UMNOCC-PEG (**Figure 12B**). After intratumoral injection, the tumor site showed a significantly enhanced CT signal (360.6 HU) compared with pre-injection (20.3 HU, **Figure 12C**), indicating that UMNOCC-PEG possessed outstanding CT imaging capability. After intravenous administration, the signal intensity at the tumor region at 12 h post-injection was approximately 4-fold higher than pre-injection, demonstrating the superior tumor enrichment capability of UMNOCC-PEG. Meanwhile, the acidic TME accelerated the decomposition of CuO_2_ and synchronously induced the Fenton-like catalytic reaction of Cu^2+^ and H_2_O_2_ to form highly toxic •OH. UMNOCC-PEG also alleviated tumor hypoxia and antioxidant capability through NO production and glutathione consumption and enhanced the efficiency of PDT and chemodynamic therapy (CDT). Furthermore, NO and ROS could produce reactive nitrogen species (RNS) to destroy DNA and facilitate the therapeutic effect (**Figure 12D-E**). Therefore, the CT image-guided gas therapy-based synergistic therapy showed significant tumor inhibition efficiency (**Figure 12F**).

In another example, Yang *et al.* fabricated X-ray-triggered NO-released multifunctional Bi-based nanotheranostic Bi-SNO NPs for CT image-guided combination therapy of RT, PTT, and gas therapy (**Figure 12G**) 175. The CT values increased linearly with increasing Bi-SNO NPs concentration after intratumoral injection, and the CT signal at the tumor region was significantly enhanced (232.21 HU) compared to pre-injection (47.52 HU), indicating that Bi-SNO NPs were an excellent CT contrast agent (**Figure 12H**). Subsequently, CT image-guided X-ray irradiation could break down the S-N bond and induce *in situ* release of NO in specific tumor regions, triggering gas therapy and RT. PTT was also realized under 808 nm NIR laser irradiation. Thus, the combination of gas therapy, RT, and PTT caused apoptosis and necrosis of tumor cells and resulted in tumor volume reduction (**Figure 12I-J**).

### Sonodynamic therapy

**Figure 13:**
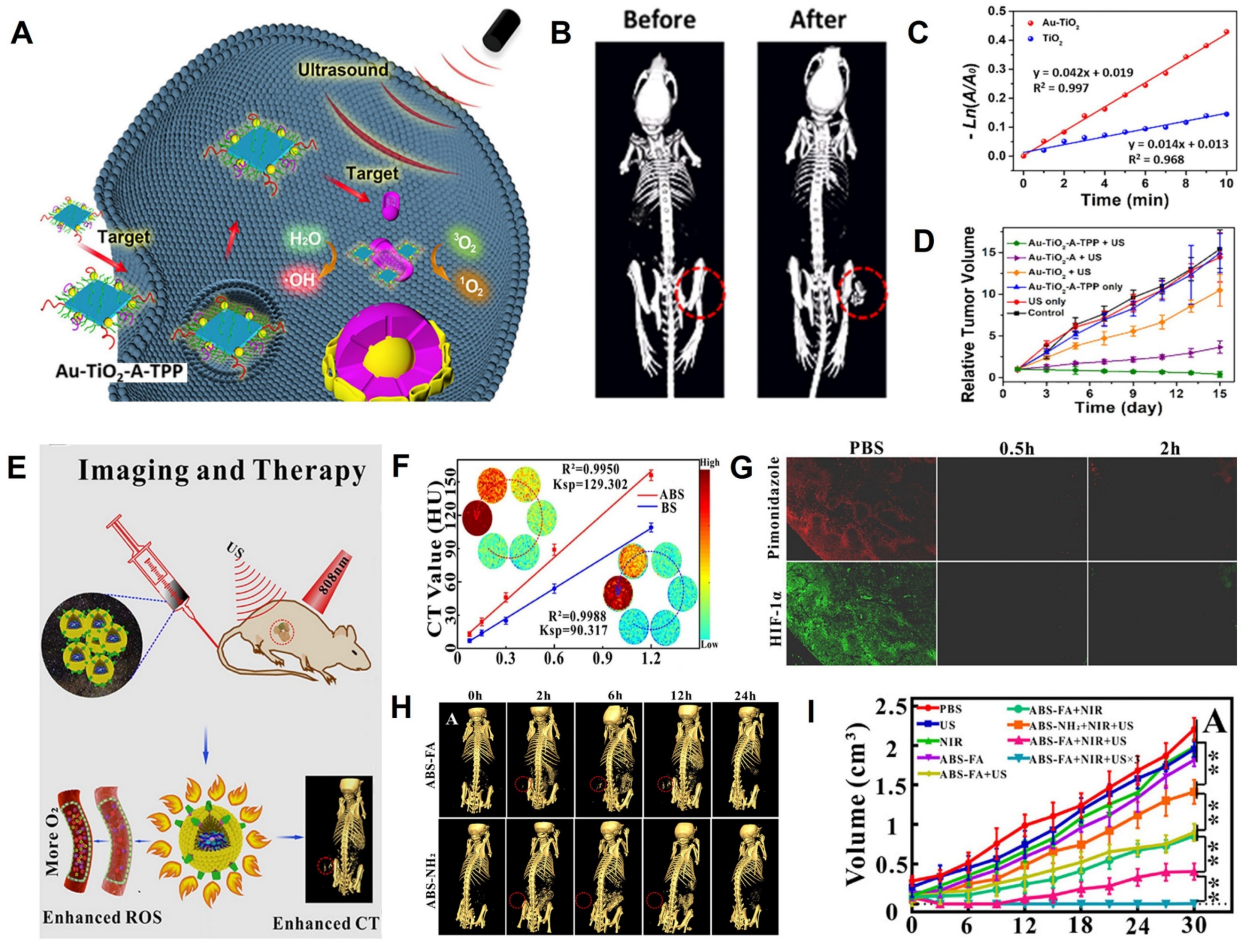
CT image-guided SDT. **(A)** Schematic illustration of dual-targeted Au-TiO_2_-A-TPP for SDT. **(B)** CT images of tumor-bearing mice at pre- and post-intravenous Au-TiO_2_-A-TPP injection. **(C)** ^1^O_2_ generation under US irradiation. **(D)** Changes in relative tumor volumes. **(E)** Proposed principle of CT image-guided therapy. **(F)** CT values of ABS and BS at different concentrations. **(G)** Immunofluorescence staining of tumor site at 0.5 h and 2 h under ABS-FA plus NIR treatment. **(H)** Changes in CT contrast with time after injection of ABS-FA or ABS. **(I)** Tumor volumes in HeLa tumor-bearing mice. **(A-D)** Adapted with permission from 182, Copyright 2019 American Chemical Society. **(E-I)** Adapted with permission from 183, Copyright 2020 American Chemical Society.

Sonodynamic therapy (SDT), which consists of low-intensity ultrasound (US) combined with sonosensitizers, has emerged as a burgeoning cancer treatment method since its development in the late 1980s. ​SDT uses sonosensitizers to absorb the US energy, producing a cavitation effect to excite O_2_ and generate ROS for killing tumor cells 176-181. Zhang *et al.* developed a dual-targeted sonosensitizer (Au-TiO_2_-A-TPP) that was assembled by TiO_2_ NSs and Au NCs modified with mitochondria-targeted triphenylphosphine (TPP) and cancer cell membrane-targeted AS1411 aptamer for CT image-guided SDT (**Figure 13A**) 182. After intravenous injection of Au-TiO_2_-A-TPP, the CT signal at the tumor region continuously increased and reached the maximum value at 24 h post-injection (**Figure 13B**), at which point the tumor region was irradiated with US. The Au-TiO2 NSs enriched in the tumor absorbed the US energy and facilitated the transfer of interfacial electrons under US irradiation, resulting in ROS production (**Figure 13C**). Consequently, the CT image-guided SDT resulted in efficient tumor growth inhibition and no recurrence (**Figure 13D**).

However, SDT depends on intratumor O_2_ concentration, and the hypoxic TME compromises SDT efficiency. Therefore, increasing the O_2_ content in the tumor region has become a major issue that must be addressed. Liu *et al.* synthesized targeting multifunctional hydrophilic nanomicelles AgBiS_2_@DSPE-PEG_2000_-FA (ABS-FA), which had significant CT imaging capability and were used for the cascade-enhanced synergistic effect of SDT and PTT (**Figure 13E**) 183. During the synergistic therapy, PTT heat could facilitate local blood circulation beneficial to alleviate the hypoxic TME. The CT values increased as a function of ABS-FA concentration, and the linear slope of ABS-FA was 43.21% higher than that of the traditional Bi_2_S_3_ NPs (**Figure 13F**) because the molar amount of metal per unit mass of AgBiS_2_ was 1.35-fold higher than Bi_2_S_3_. Therefore, ABS-FA possessed a stronger X-ray attenuation to enhance the CT imaging capability. The reoxygenation of tumor cells was observed within 2 h after NIR laser irradiation because the photothermal effect could continuously alleviate hypoxia (**Figure 13G**). After intravenous injection of ABS-FA, CT signal intensity at the tumor site gradually increased over time and peaked at 6 h post-injection (**Figure 13H**), which was chosen as the starting time point for the ultrasound-photothermal synergistic therapy. The tumor volume was significantly inhibited 30 days after treatment (**Figure 13I**), suggesting that CT image-guided SDT-based therapy can open up new ways for clinical applications.

### Gene therapy and microwave thermal therapy

**Figure 14:**
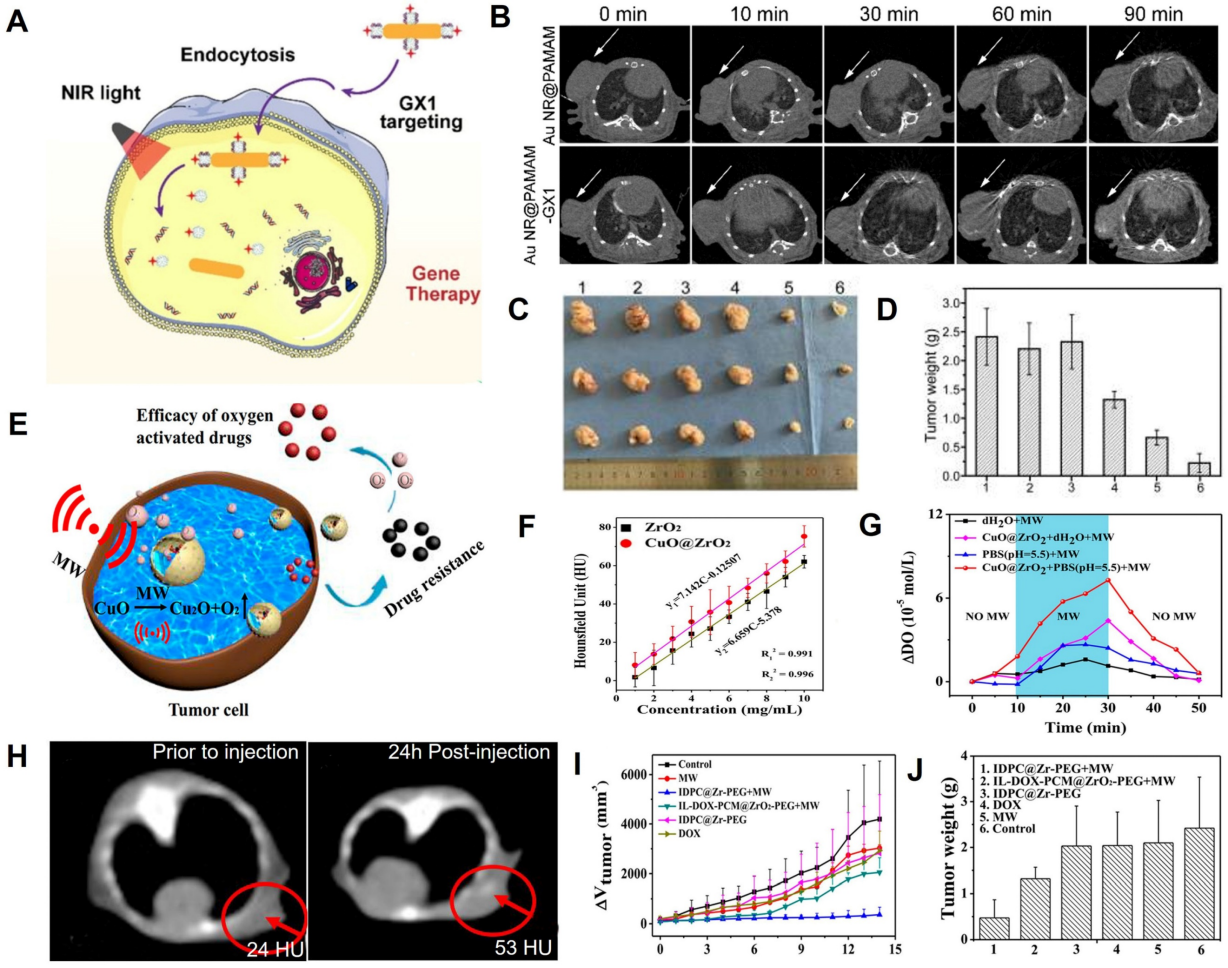
CT image-guided GT and MWTT. **(A)** Schematic illustration of FAM172A-mediated gene therapy. **(B)** Changes of CT contrast with time after injection. **(C)** Photographs of HCT-8 tumors at 14 days after different treatments. **(D)** Corresponding tumor weights. **(E)** Schematic illustration of IDPC@Zr-PEG for cancer treatment. **(F)** CT values of IDPC@Zr nanocomposites at different concentrations. **(G)** O_2_-production capability of IDPC@Zr under MW irradiation. **(H)** CT images of the tumor at per- and 24 h post-injection. **(I)** Tumor volume and **(J)** Tumor weight in different treatment groups at 14 days post-treatment. **(A-D)** Adapted with permission from 188, Copyright 2021 BioMed Central. **(E-J)** Adapted with permission from 190, Copyright 2018 American Chemical Society.

Gene therapy (GT) can achieve long-term effects by introducing specific genes into target cells to repair defective genes or promote specific cellular functions 184, 185. For example, FAM172A, as a functional gene, can inhibit the proliferation and accelerate apoptosis of colon cancer cells 186. However, FAM172A is readily degraded by enzymes in the blood, so carrier protection is required to deliver FAM172A to the target region 187. Therefore, Cui *et al.* designed the circular heptapeptide GX1-targeting multifunctional nanoagents (AuNR@PAMAM-GX1/FAM172A) consisting of dendrimer-modified AuNRs and FAM172A for CT image-guided GT/PTT of colon cancer cells (HCT-8 cells) (**Figure 14A**) 188. After intravenous administration of AuNR@PAM-GX1/FAM172A to HCT-8 tumor-bearing nude mice, the CT signal at the tumor region gradually increased over time and peaked at 90 min post-injection, demonstrating the maximum enrichment of the AuNR@PAM-GX1/FAM172A in the tumor at that time point (**Figure 14B**). Based on CT imaging information, NIR irradiation at the tumor region was performed at 90 min post-injection to produce a photothermal effect, while the FAM172A was released for the combination of PTT and GT (**Figure 14C-D**).

Microwave (MW) thermal therapy (MWTT) is an emerging treatment method that promotes rapid heating and induces apoptosis in the tumor region through MW irradiation 189. Meng *et al.* synthesized PEGylated IL-DOX-PCM-CuO@ZrO_2_ multifunctional nanocomposites (IDPC@Zr-PEG) to upregulate tumor reoxygenation by utilizing CuO NPs to produce O_2_ under MW irradiation in TME for improving the effect of combined cancer therapy of CT image-guided MWTT and chemotherapy (**Figure 14E**) 190. ​The increase in CT values as a function of IDPC@Zr-PEG concentration due to the high atomic number of Zr demonstrated that IDPC@Zr-PEG possessed excellent CT imaging capabilities and can be used for real-time monitoring of tumor treatment. (**Figure 14F**). The MW irradiation of CuO enabled electron transfer and oxygen generation (**Figure 14G**) to alleviate hypoxia. CT values of the tumor region increased from 24 HU at pre-injection to 53 HU 24 h after intravenous injection of IDPC@Zr-PEG (**Figure 14H**). Based on the CT imaging information, the tumor was irradiated by MW at 24 h post-injection, and the tumor inhibition rate reached 92.14% after 14 days of treatment (**Figure 14I-J**). The results indicated that the CT image-guided MWTT provided a promising approach for clinical cancer therapy.

## Conclusion and Outlook

​CT imaging has evolved as an important tool for preclinical research and clinical applications. CT contrast agents are functional substances that can provide biomedical information by correlating imaging signals with molecular states or biological events *in vivo*, a prerequisite, and core technology for implementing accurate molecular imaging. Iodinated contrast agents, which account for 55% of total CT contrast agents, are widely used for diagnosing diseases and improving the resolution of CT images in soft tissues, particularly for identifying cancerous and normal tissues. However, iodinated contrast agents suffer from inherent drawbacks such as low contrast, short cycle times, and single function, making it challenging to meet the increasing clinical demands. Nanomaterial-based CT contrast agents have attracted much attention due to their various advantages, including homogeneous morphology, controllable size, high dispersion, excellent stability, and ease of functionalization. In particular, designing versatile nanomaterial-based CT contrast agents with comprehensive diagnostic and therapeutic capabilities has become a research hot spot.

Herein, we provide a comprehensive and up-to-date overview of recently reported studies on available CT contrast agents and their biomedical applications. CT contrast agents, such as Au-based nanomaterials, Bi-based nanomaterials, Ln-based nanomaterials, TM-based nanomaterials have been described. Regardless of various types, CT contrast agents can be used to CT image-guided therapy, including PTT, PDT, chemotherapy, gas therapy, RT, SDT, immunotherapy, starvation therapy, and synergistic therapy, among others.

Current research on the available CT contrast agents for cancer theranostics focuses on two main aspects: 1) designing multimodal imaging contrast agents and leveraging their complementary advantages to improve disease diagnosis accuracy and 2) integrating multiple functions into a single contrast agent, enabling the integration of diagnosis and treatment. Although there have been various reports on CT contrast agents in recent years, challenges remain in their design, large-scale preparation, cost, biocompatibility, potential toxicity, sensitivity, and specificity. Efforts should be devoted to addressing the following challenging issues:

1) Developing endogenous biomarker-responsive CT contrast agents to improve sensitivity and specificity. Contrast agents are a central theme in CT imaging and are directly related to imaging quality. Therefore, harnessing biomarkers in the tumor microenvironment as stimulus-response units to design intelligent CT contrast agents is central to enhancing imaging contrast and improving the specificity and accuracy of disease diagnosis.

2) ​Although using CT contrast agents can enhance imaging contrast and improve diagnosis accuracy, only a few are currently available for use in the clinic due to their poor biocompatibility and potential toxicity. Optimizing the biological properties of contrast agents, such as stability, biocompatibility, toxicity, and targetability, to broaden their applications would be a promising research direction.

3) “All-in-one” theranostic nanoplatforms for disease diagnosis and treatment have received increasing attention due to the integration of multiple functions and are expected to have the best chance of clinical success. Researchers should focus on utilizing contrast agents as an “all-in-one” nanotheranostic platform to allow real-time diagnosis and synchronous treatment. For example, AuNPs, with controllable size, easy surface modification, perfect radiosensitization capability, tunable optical properties, and high X-ray attenuation, can simultaneously realize multimodal (CT, MR, PA) image-guided synergistic treatment (RT, PTT) 191.

## Figures and Tables

**Table 1:** Commercial CT contrast agents and their properties

Common name	Commercial name	Properties	Molecular formula	Advantages	Disadvantages
Meglumine diatrizoate	Angiografin^®^	Ionic monomers (hyperosmolality)	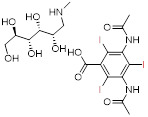 C_18_H_26_I_3_N_3_O_9_	Low cost, applications in ureterography and salpingography.	Anaphylaxis, nephrotoxicity, and local stimulation not recommended for angiography
Iopromide	Ultravist^®^	Nonionic monomers (secondary hyperosmolality)	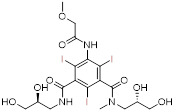 C_18_H_24_I_3_N_3_O_8_	High solubility and hydrophilicity, permission for angiography.	Nephrotoxicity, lactic acidosis
Iohexol	Omnipaque^®^	Nonionic monomers (secondary hyperosmolality)	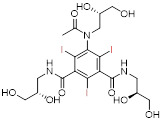 C_19_H_26_I_3_N_3_O_9_	Low viscosity and high safety, permission for myelography and ventriculography.	Anaphylaxis, nephrotoxicity
Iodixanol	Visipaque^®^	Nonionic dimer (isoosmolality)	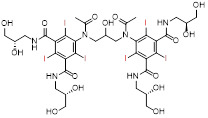 C_35_H_44_I_6_N_6_O_15_	Minimal nephrotoxicity, applicable to patients with renal insufficiency.	High cost

**Table 2:** List of contrast agents for CT imaging in biomedicine

Element and atomic number	K-edge energy [keV]	X-ray mass attenuation coefficient at 100 keV [cm^2^ g^- 1^]	Representative contrast agents	Advantages	Disadvantages	Therapeutic application	References
I (53)	33.2	1.94	Iopromide, Iodixanol, Iohexol, Meglumine diatrizoate	Low-cost, large-scale production, wide application in clinical practice	Low contrast, short retention time *in vivo*, allergic reaction, nephrotoxicity	NA	8
**Lanthanide elements**							
Ce (58)	40.4	2.45	CeO_2_ NPs	High colloidal stabilities, rich in resources	Low yield, short circulation times, nephrotoxicity	PTT, PDT, RT, starvation therapy, gas therapy	71
Eu (63)	48.5	3.04	Eu_2_O_3_ NPs				70
Gd (64)	50.2	3.11	Gd_2_O_3_ NPs				68
Ho (67)	55.6	3.49	NaHoF_4_ NPs				74
Er (68)	57.5	3.63	NaErF_4_ NPs				75
Yb (70)	61.3	3.88	NaYbF_4_ NPs				77
Lu (71)	63.3	4.03	NaYF_4_:Nd^3+^@NaLuF_4_				83
**Transition metal elements**							
Hf (72)	65.3	4.15	HfO_2_ NPs	Physiological inertness, easy of functionalization	Complex syntheses, relatively high-cost	PTT, RT, chemotherapy	89
Ta (73)	67.4	4.30	TaOx NPs				92
W (74)	69.5	4.44	RbxWO_3_ NRs				97
Re (75)	71.7	4.59	ReS_2_ NSs				99
Ir (77)	76.1	4.86	Ir-Ag-IrO_2_ NPs				101
Pt (78)	78.4	4.99	Pt Spirals				103
**Gold element**							
Au (79)	80.7	5.16	AuNSp, AuNSs, AuNRs, AuNCs, AuNShs	Mature preparation methods, easy surface modification, high biocompatibility	High-cost, hepatotoxicity	PTT, PDT, RT, SDT, GT, immunotherapy, chemotherapy	14-27
**Bismuth element**							
Bi (83)	90.5	5.74	Bi_2_S_3_ NPs, Bi_2_Se_3_ NPs, Bi_2_O_3_ NPs, Cu_3_BiS_3_ NPs	High X-ray attenuation capability, relatively low toxicity, low-cost	Poor solubility, limited contrast effect at low X-ray tube voltages (< 80 keV)	PTT, PDT, RT, SDT, gas therapy, chemotherapy	56-62, 64-65

PPT: photothermal therapy; PDT: photodynamic therapy; RT: radiotherapy; SDT: sonodynamic therapy, GT: gene therapy; MWTT: microwave thermal therapy.

## Abbreviations

ADCC: antibody-dependent cell-mediated cytotoxicity
AuNCs: gold nanocages
AuNPs: gold nanomaterials
AuNPrs: gold nanoprisms
AuNPTs: gold nanoplates
AuNRs: gold nanorods
AuNSs: gold nanostars
AuNShs: gold nanoshells
AuNSps: gold nanospheres
BiNPs: bismuth nanoparticles
BSA: bovine serum albumin
CDT: chemodynamic therapy
CPP: cell-penetrating peptide
CSS: core-shell-shell
CT: computed tomography
DOX: doxorubicin
DXL: docetaxel
EGFR: epidermal growth factor receptor
EMI: electric and musical industries
FA: folic acid
FR: folate receptor
GOD: glucose oxidase
GIT: gastrointestinal tract
GT: gene therapy
HA: hyaluronic acid
HIF: hypoxia-inducible factor
HU: Hounsfield unit
ICB: immune checkpoint blockade
LSPR: localized surface plasmon resonance
MPE: maximum permissible exposure
MRI: magnetic resonance imaging
MW: microwave
MWTT: microwave thermal therapy
NIR: near infrared
NGs: nanogels
NPs: nanoparticles
NRs: nanorods
NSs: nanosheets
PA: photoacoustic
PAA: polyacrylic acid
PCE: photothermal conversion efficiency
PCL: poly-ε-caprolactone
PDA: polydopamine
PDL1: programmed death ligand 1
αPDL1: programmed death ligand 1 antibody
PDT: photodynamic therapy
PEG: polyethylene glycol
PEI: polyethylenimine
PFH: perfluorohexane
PL: photoluminescence
PSs: photosensitizers
PTAs: photothermal agents
PTT: photothermal therapy
PVA: polyvinyl alcohol
PVCL: poly(N-vinylcaprolactam)
PVP: polyvinylpyrrolidone
RNS: reactive nitrogen species
RT: radiotherapy
SDT: sonodynamic therapy
SER: sensitization enhancement ratio
SPR: surface plasmon resonance
TM: transition metal
TME: tumor microenvironment
TPP: triphenylphosphine
US: ultrasound
